# QRICH1 regulates ATF6 transcription to affect pathological cardiac hypertrophy progression

**DOI:** 10.1186/s10020-025-01241-2

**Published:** 2025-05-13

**Authors:** Lihui Zhang, Hongping Chen, Guangmei Zou, Wenjuan Jia, Haibin Dong, Chunxiao Wang, Hua Wang, Yugang Liu, Da Teng, Bowen Xu, Lin Zhong, Lei Gong, Jun Yang

**Affiliations:** 1 Medical College, Qingdao University, Qingdao, Shandong China; 2 Present Address: Department of Cardiology, Affiliated Yantai Yuhuangding Hospital of Qingdao University, Yantai, Shandong China; 3 Department of Cardiac Surgery, Affiliated Yantai Yuhuangding Hospital of Qingdao University, Yantai, Shandong China; 4 Present Address: Department of Cardiology, The Affiliated Hospital of Xuzhou Medical University, Xuzhou, China

**Keywords:** Cardiac remodeling, Heart failure, Endoplasmic reticulum stress, Pressure overload

## Abstract

**Background:**

Many studies have shown that pathological cardiac hypertrophy is associated with active endoplasmic reticulum (ER) stress. Glutamine-rich protein 1 (QRICH1), as a transcriptional regulator, belongs to the caspase recruitment domain (CARD)-containing gene family. QRICH1 has been shown to influence the outcomes of endoplasmic reticulum stress by regulating the transcription of proteostasis-related genes. In this study, we explored the role of QRICH1 in pathological cardiac hypertrophy.

**Methods:**

We observed an increased expression of QRICH1 in the hearts of humans and mice with left ventricular hypertrophy (LVH). To assess the functional impact in this context, we employed gain- and loss-of-function approaches, using AAV9 injections to establish cardiac-specific QRICH1 knockdown or overexpression models in transverse aortic constriction (TAC) or isoproterenol (ISO)-induced cardiac hypertrophy.

**Results:**

Our data indicated that cardiomyocyte-specific knockdown of QRICH1 alleviated the hypertrophic phenotype in response to TAC or ISO injection. However, overexpression of QRICH1 exacerbated cardiac hypertrophy, remodeling, dysfunction, cell apoptosis, and inflammatory responses. Mechanistically, we demonstrated that ATF6 was significantly enriched by QRICH1 in cardiomyocytes treated with ISO using RNA-seq combined with CUT&TAG analysis. ChIP-qPCR and luciferase assays further confirmed that ATF6 is a target gene of QRICH1 in cardiomyocytes under growth stimulation. Knockdown of QRICH1 in cardiomyocytes blocked ISO-mediated induction of ATF6, activation of mTORC1, and cellular growth. And all of the above was restored by the overexpression of ATF6.

**Conclusions:**

QRICH1 plays a pivotal role in cardiac hypertrophy by regulating ATF6, and QRICH1 may be a potential new therapeutic target for pathological cardiac hypertrophy.

**Supplementary Information:**

The online version contains supplementary material available at 10.1186/s10020-025-01241-2.

## Background

ER homeostasis plays a crucial role in synthesizing essential proteins for cardiac myocytes, including calcium-handling proteins, receptors, secreted hormones, stem cell homing factors, membrane proteins, and growth factors to maintain heart function (Glembotski 2012; Martin et al. 2023; Neufeldt et al. 2024). The disruption of ER homeostasis, commonly known as ER stress, plays a critical role in cardiac function and pathology (Ren et al. 2021; Lemmer et al. 2021; Pakos-Zebrucka et al. 2016; Omidkhoda et al. 2019). ER stress and the unfolded protein response (UPR), especially through the double-stranded RNA-activated protein kinase R-like endoplasmic reticulum kinase (PERK) and eif2α-ATF4-CCAAT-CHOP signaling pathways, are implicated in the onset of cardiac hypertrophy and heart failure (Yao et al. 2017). Prolonged ER stress, triggered by conditions like pressure overload, plays a critical role in the progression from cardiac hypertrophy to failure. This is likely due to its influence on inflammation and apoptosis within cardiac myocytes (Okada et al. 2004; Iurlaro and Munoz-Pinedo 2016; Gorman et al. 2012).

Caspase recruitment domain (CARD), a well-known protein–protein interaction module, mediates important cellular signaling events related to a variety of human diseases, including cancer, neuro-degenerative diseases and immune disorders (Park 2019). QRICH1, a member of the CARD-containing gene family, serves as a key regulator of a unique transcriptional modulation that orchestrates cellular stress responses, which is pivotal in regulating protein synthesis and secretion under various states of homeostasis and pathology (Bouchier-Hayes and Martin 2002). QRICH1 is expressed in various organs and can participate in apoptosis, inflammation, and immune responses through protein–protein interactions (Kumble et al. 2022). A recent study about the stress response of mouse intestinal epithelial cells to ER stress showed that the translation of QRICH1 could be promoted by the phosphorylation of eIF2α in the PERK pathway. QRICH1 may affect the ER stress process through the transcriptional regulation of protein balance and determine the fate of cells in the pathological ER stress response (You et al. 2021).

QRICH1 plays a vital role in the development of humans and rodents. In humans, variants of QRICH1 can lead to developmental defects, abnormal longitudinal bone formation, or neurodevelopmental disorders (Kumble et al. 2022). In rodents, QRICH1 variants are associated with conditions such as cleft palate and renal abnormalities (Baruch et al. 2021). Recent research has revealed a significant link between QRICH1 and heart disease. Mutations in the QRICH1 gene, such as the Qrich1^b2b2404Clo^ mutation in mice, can result in heart-related phenotypes including ventricular septal defects and incomplete left ventricular compaction (San Agustin et al. 2016; Li et al. 2015). Furthermore, QRICH1 has been found to interact with myosin heavy chain, a crucial component of thick filaments in muscle sarcomeres (Luck et al. 2020). This interaction suggests that QRICH1 may play a role in regulating the pathogenesis of myocardial hypertrophy.

In this study, we confirmed that QRICH1 could exacerbate hypertrophy, fibrosis, apoptosis, inflammation, and cardiac dysfunction in cardiac hypertrophy models. At least in part, this effect is attributed to QRICH1 targeting the ATF6-mTOR pathway. Therefore, QRICH1 may play a pivotal role in orchestrating protein synthesis and folding, as well as sustaining protein homeostasis, by modulating the transcription of ATF6 during cardiac hypertrophy.

## Methods

Data supporting the findings of this study are available from the corresponding author upon request. The detailed and expanded methodology used in this study are located in the Supplemental Materials online.

### Statistical analysis

Continuous variables were represented as mean ± standard error of the mean (SEM). The Shapiro–Wilk test was used to determine the normal distribution of data, while the Brown-Forsythe test was employed to assess the homogeneity of variances. When data were normally distributed, an unpaired two-tailed Student's t-test was used to compare means between two groups; otherwise, the non-parametric Mann–Whitney U test was applied. Analysis of variance (ANOVA) was followed by post-hoc analysis using either the Bonferroni or Tukey method to adjust for multiple comparisons. If not, the non-parametric Kruskal–Wallis test and Dunn's multiple comparison test were used. Unless otherwise specified, a two-sided significance level of 0.05 was set. All data were analyzed and visualized using GraphPad Prism (version 9.5.1).

## Results

### Increased cardiac QRICH1 expression under hemodynamic stress

**Fig. 1:**
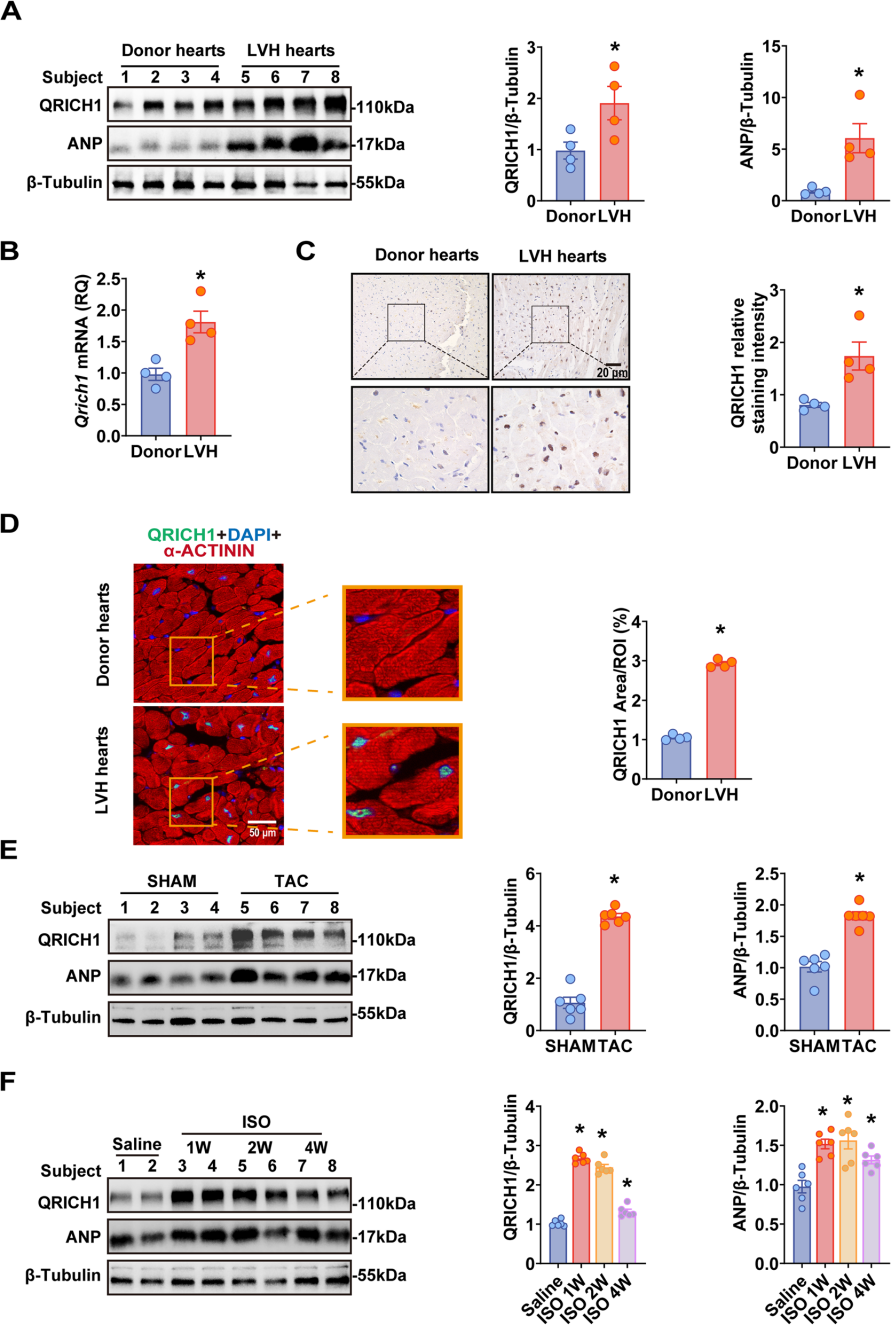
QRICH1 expression and activation are increased in human and mouse hearts with LVH (left ventricular hypertrophy). **A**, Western blots (left) and quantification (right) of QRICH1, ANP levels in normal donor hearts (*n* = 4) and hearts collected from LVH patients (*n* = 4). **B**, qRT-PCR analysis of *QRICH1* mRNA levels in normal or LVH human hearts (*n* = 4 samples per group). **C**, Representative images (left) and quantification (right) of QRICH1 immunohistochemistry from human hearts. **D**, Immunofluorescence images (left) and quantification (right) of α-actinin (red), QRICH1 (green) and DAPI (4’,6-diamidino-2-phenylindole; blue) staining in the LV from normal donor and LVH patients. *n* = 4 samples per group. **E**, Western blots (left) and quantification (right) of QRICH1, ANP levels in mouse hearts at 4 weeks after sham operation or transverse aortic constriction (TAC) surgery (*n* = 6 mice per group). **F**, Western blots (left) and quantification (right) of QRICH1, ANP expression levels in mouse hearts treated with phosphate-buffered saline (PBS) or isoproterenol (ISO; 1 μmol/L) injection for 1, 2 and 4 weeks (*n* = 6 mice per group). *P < 0.05 compared with respective controls. Data are presented as mean ± SEM. **A**, Mann–Whitney *U* test; **B** through **E**, unpaired two-tailed Student’s *t*-test; F, one-way ANOVA with post-hoc multiple comparisons

To investigate the potential impact of QRICH1 on the development of cardiac hypertrophy and heart failure, we initially analyzed the expression levels of QRICH1 in left ventricle samples from patients with left ventricular hypertrophy (LVH) compared to normal donor heart samples. Detailed information on the human heart samples is provided in (Supplementary Table 1). We found that both QRICH1 mRNA and protein levels were significantly increased in LVH samples, indicating that elevated QRICH1 expression may be associated with the development of cardiac hypertrophy and failure. Specifically, QRICH1 protein content was elevated 2.0-fold in LVH samples compared to those from normal donors, representing a significant difference (*P* < 0.05). Additionally, in LVH samples, the level of ANP—a biomarker associated with heart failure—was significantly elevated (Fig. 1A, B), suggesting the presence of heart failure-related stress in LVH. To further verify the subcellular localization of QRICH1 in humans, immunohistochemical staining revealed a higher concentration of QRICH1 in the nuclei of cardiomyocytes from patients with failing hearts compared to those from healthy individuals, suggesting a nuclear localization of QRICH1 in the context of heart failure (Fig. 1C). This was corroborated by immunofluorescent staining, confirming the nuclear localization of QRICH1 in cardiomyocytes (Fig. 1D).

To test whether QRICH1 is responsive to pathological cardiac hypertrophy, we conducted a study using a mouse model subjected to pressure overload induced by transverse aortic constriction (TAC) and a catecholamine-induced model injected with isoproterenol (ISO). At 4 weeks post-TAC, we observed changes that were consistent with those observed in human tissues. Compared with sham-operated hearts, the expression levels of QRICH1 in TAC hearts increased by 1.7-fold at the protein level (Fig. 1E). Similarly, compared to PBS-treated hearts, the expression levels of QRICH1 were significantly increased in mouse hearts injected with ISO from weeks 1 to 4 (Fig. 1F). Additionally, after treating neonatal rat cardiomyocytes (NRCMs) with ISO (1 μmol/L) for 24, 48, and 72 h to induce hypertrophy, we observed a time-dependent upregulation of QRICH1 protein expression in cardiomyocytes (Supplementary Fig. 1A). We also observed that under ISO treatment, the expression of QRICH1 in cardiomyocytes was higher compared to non-cardiomyocytes (Supplementary Fig. 1B). Taken together, these data suggest that hemodynamic overload leads to increased expression and activation of QRICH1.

### QRICH1 deficiency alleviates cardiac hypertrophy and Cardiac remodelling in TAC mice

**Fig. 2:**
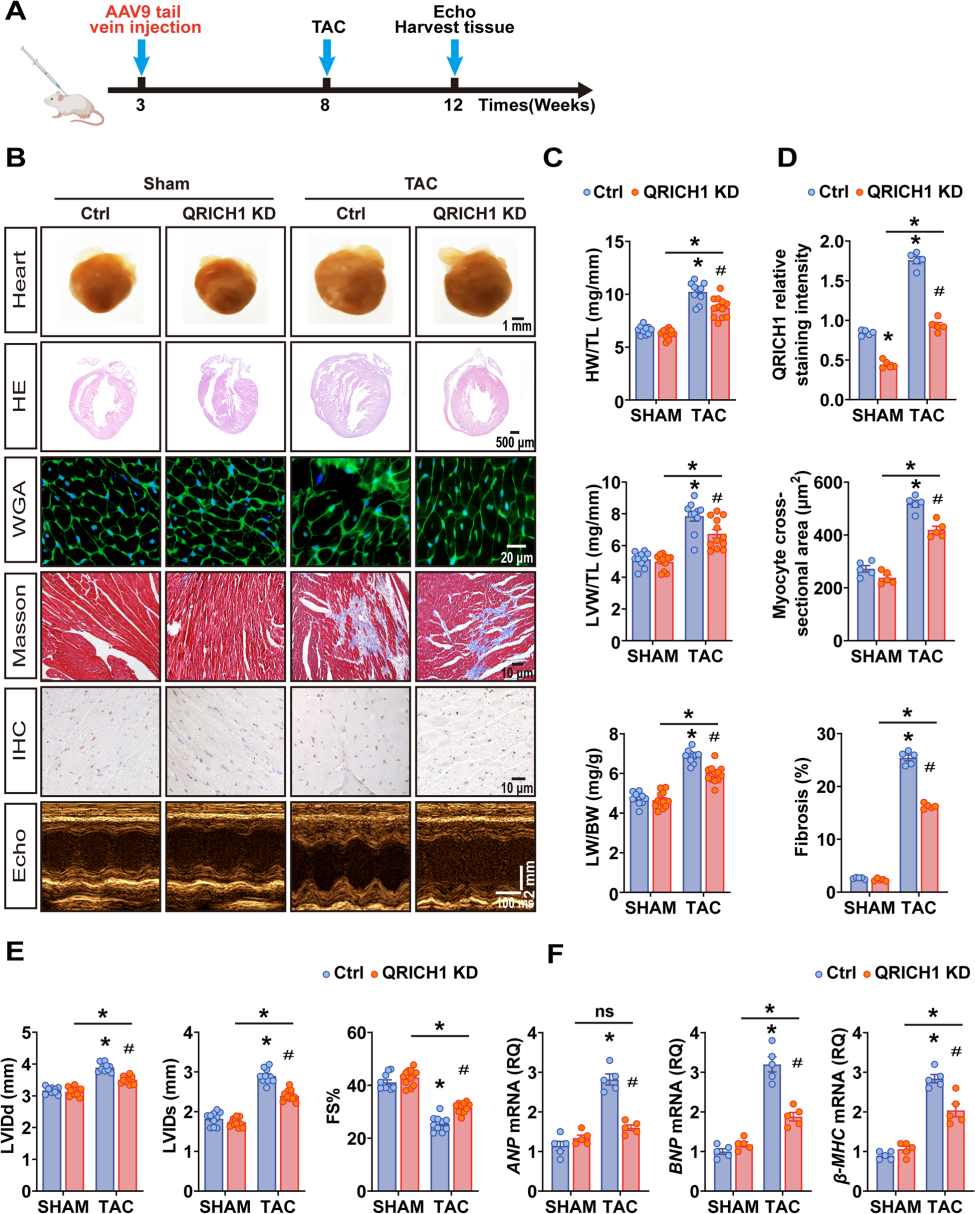
Cardiac-specific knockdown of QRICH1 alleviates pathological cardiac hypertrophy and remodelling. **A**, Treatment regimen for Ctrl and QRICH1 KD mice in vivo studies. Adeno-associated virus of serotype 9 (AAV9) containing either GFP or shQRICH1 construct targeting cardiomyocytes was injected into mice at a concentration of 5 × 10^11^ (genome copies/mL) for 5 weeks. The mice were then subjected to TAC for 4 weeks. **B**, Representative gross morphology of mouse hearts (the top row, scale bars = 1 mm), cross-sections of the heart stained with Hematoxylin and Eosin (the second row, scale bars = 500 μm), cell boundaries stained with wheat germ agglutinin (the third row, scale bars = 20 μm), LV fibrosis stained with Masson's trichrome (the forth row, scale bars = 10 μm), LV QRICH1 expression determined by immunohistochemistry (the fifth row, scale bars = 10 μm), M-mode echocardiography images of the LV chamber in QRICH1 KD and Ctrl littermate mice subjected to sham or TAC surgery (the bottom row, scale bars = 2 mm). **C**, The ratio of heart weight to tibia length (HW/TL), left ventricle weight to tibia length (LVW/TL), lung weight to body weight (LW/BW) (*n* = 10,12,10,12 mice from left to right). **D**, Statistical results for QRICH1 immunohistochemistry expression, quantification of cell cross-sectional area and myocardial interstitial collagen (*n* = 5 mice per group). **E**, Echocardiographic measurements of LV end-diastolic internal diameter (LVIDd), LV end-systolic internal diameter (LVIDs), and fractional shortening (FS) in Ctrl and QRICH1 KD mice 4 weeks after sham or TAC surgery (*n* = 10–12 mice per group). **F**, Measurement levels of myocardial hypertrophy-associated transcripts ANP (atrial natriuretic peptide), BNP (brain natriuretic peptide), β-MHC (β myosin heavy chain) (*n* = 5 mice per group). *P < 0.05 compared to Ctrl/SHAM group, or the value shown by the bar. ^#^*P* < 0.05 compared to Ctrl/TAC group. Data are presented as mean ± SEM. **C**, **D**, **E** and **F**, 2-way ANOVA followed by Bonferroni post-test or Tukey post-test

We aimed to investigate whether QRICH1 contributes to cardiac hypertrophy. Considering QRICH1 is upregulated in hypertrophied hearts, we hypothesized that hypertrophic stimuli induce cardiomyocyte hypertrophy through the upregulation of QRICH1. To test this, we employed adeno-associated virus 9 (AAV9) to deliver short hairpin RNAs (shRNAs) targeting QRICH1 for knockdown (QRICH1 KD) in cardiac myocytes, and TAC was performed 5 weeks post-injection (Fig. 2A, Supplementary Fig. 2A) (Yuan et al. 2023; Remes et al. 2021; Su et al. 2023; Xie et al. 2023). Compared to mice injected with AAV9-Ctrl, AAV9-mediated delivery of QRICH1 shRNAs, using a cardiac-specific cTNT promoter, effectively reduced QRICH1 mRNA or protein expression in cardiac myocytes isolated from QRICH1 KD mice, without affecting QRICH1 mRNA levels in non-cardiac cells or the liver (Supplementary Fig. 2B, C). Under basal conditions, hearts from QRICH1 KD mice were macroscopically indistinguishable from those of control mice (Fig. 2B). However, compared with control mice, QRICH1 KD mice exhibited a significantly ameliorated phenotype, with the heart weight to tibia length ratio reduced by 15% and the left ventricular weight to tibia length ratio decreased by 14% 4 weeks after TAC (Fig. 2C). Additionally, QRICH1 KD mice exhibited a decrease in the cross-sectional area of cardiomyocytes and a reduction in cardiac fibrosis (Fig. 2B, D). While no significant differences in cardiac structure and function were observed under baseline measurements, echocardiography indicated that, after TAC surgery, QRICH1 KD mice exhibited a decrease in LV internal diameter, LV septum thickness, LV posterior wall thickness, and the ratio of early mitral inflow velocity to early mitral annular tissue velocity. Furthermore, these mice showed an increase in fractional shortening and ejection fraction (Fig. 2E; Supplementary Fig. 2D; Supplementary Table 2). Moreover, in QRICH1 KD mice, the lung weight to tibia length ratio decreased, indicating alleviated pulmonary congestion, and there was also a reduction in the expression of the hypertrophic markers (atrial natriuretic peptide, brain natriuretic peptide, and β-myosin heavy chain) post-TAC (Fig. 2C, F). Consistently, ELISA analysis detected a reduction in the levels of TNF-α, IL-1β, and IL-6 in the circulation of KD mice, indicating the inhibition of TAC-induced inflammation (Supplementary Fig. 2E).

To confirm the protective role of QRICH1 against cardiac hypertrophy, we employed an alternative model of pathological myocardial hypertrophy induced by ISO injection (Supplementary Fig. 3A). Compared with corresponding parameters in control mice, QRICH1 KD mice also exhibited blunted myocardial hypertrophy, remodeling and inflammatory response (Supplementary Fig. 3B-I; Supplementary Table 3). Collectively, these data suggest that cardiomyocyte-specific knockdown of QRICH1 can prevent the occurrence of pathological cardiac hypertrophy.

### Overexpression of QRICH1 aggravates cardiac hypertrophy and failure induced by TAC

Next, we sought to determine whether QRICH1 overexpression in the heart could exacerbates TAC myocardial hypertrophy. We employed the gain-of-function approach to inject AAV9 encoding QRICH1 into mice prior to TAC surgery (QRICH1 OE mice) (Supplementary Fig. 4A). After 5 weeks, AAV9-induced QRICH1 overexpression led to a 5.1-fold increase in QRICH1 protein levels in mouse cardiomyocytes (Supplementary Fig. 4B). QRICH1 OE mice did not exhibit significant morphological or pathological cardiac abnormalities under basal conditions.

In contrast to the results observed in QRICH1 KD mice, overexpression of QRICH1 significantly exacerbated TAC-induced pathological cardiac hypertrophy, as evidenced by a significant increase in HW/TL, LVW/TL, and LW/BW ratios, along with an increase in cardiomyocyte cross-sectional area and interstitial fibrosis (Supplementary Fig. 4C-E). Consistent with these findings, QRICH1 OE mice exhibited increased internal diameters and reduced cardiac function compared to control mice, as measured by echocardiography analysis (Supplementary Fig. 4F; Supplementary Table 4). In parallel, the levels of hypertrophic markers and inflammatory markers were significantly increased in OE mice in response to TAC, suggesting that QRICH1 overexpression exacerbated ventricular remodeling and cardiac dysfunction (Supplementary Fig. 4G, H). Thus, these results suggest that overexpression of QRICH1 in the heart does not cause baseline cardiac abnormalities, but significantly renders the heart more susceptible to stress-induced pathological cardiac remodeling.

### QRICH1 promotes ISO-induced cardiomyocyte hypertrophy in vitro

**Fig. 3:**
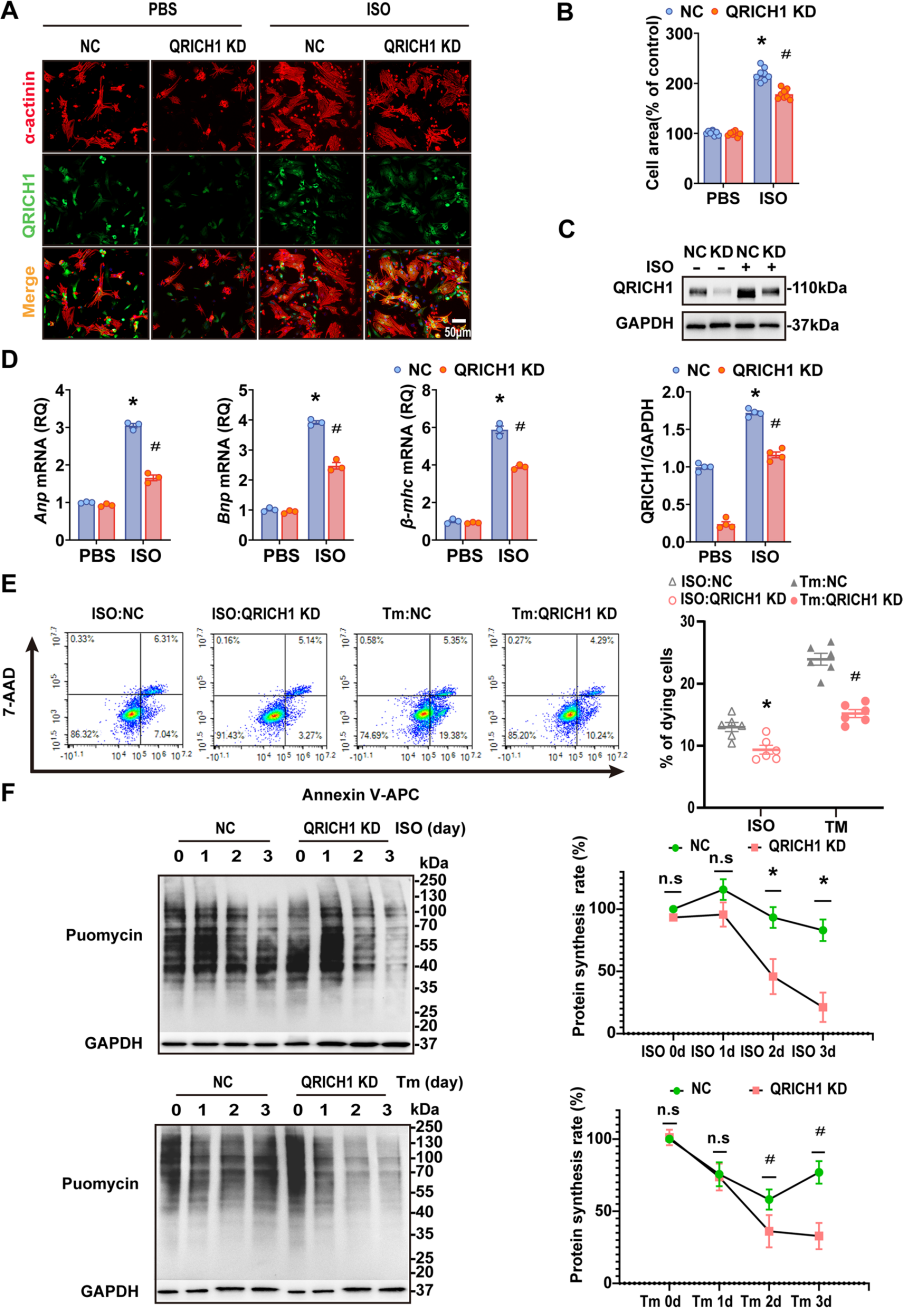
Knockdown of QRICH1 protects neonatal rat primary cardiomyocytes from ISO-induced cardiomyocyte hypertrophy. **A**, Representative immunofluorescence images of α-actinin (red), QRICH1 (green) and DAPI (4’,6-diamidino-2-phenylindole; blue) staining in Neonatal rat cardiomyocytes (NRCMs) infected with adenoviruses harboring shCtrl or shQRICH1 and treated with phosphate-buffered saline (PBS) or isoproterenol for 48 h. Scale bar = 50 μm. **B**, Quantification of relative cell surface areas of NRCMs infected with indicated adenoviruses in response to PBS or ISO treatment (*n* > 100 cells per group; *P < 0.05 compared to PBS/NC group; ^#^*P* < 0.05 compared to ISO/NC group). **C**, Western blots (upper) and quantification (lower) of QRICH1 expression in NRCMs infected with AdGFP or AdshQRICH1 (*n* = 4 independent experiments; *P < 0.05 compared to PBS/NC group; ^#^*P* < 0.05 compared to ISO/NC group). **D**, Relative mRNA levels of ANP (atrial natriuretic peptide), BNP (brain natriuretic peptide), β-MHC (β myosin heavy chain) in AdGFP- or AdshQRICH1- infected NRCMs 48 h after PBS or ISO treatment (*n* = 3 independent experiments; *P < 0.05 compared to PBS/NC group; ^#^*P* < 0.05 compared to ISO/NC group). **E**, Representative fluorescence-activated cell sorting (FACS) analysis (left) and Measurement of dying (7-AAD or Annexin V positive) cells treated with isoproterenol (ISO) or tunicamycin (Tm) for 72 h (*n* = 6 independent experiments; *P < 0.05 compared to ISO/NC group; ^#^*P* < 0.05 compared to TM/NC group). **F**, Western blots analysis of the rate of puromycin incorporation in NRCMs following pulse labeling after treatment with Tm or ISO for the indicated times and quantification of the intensity of the anti-puromycin signals (*n* = 3 independent experiments; *P < 0.05 compared to ISO/NC group; ^#^*P* < 0.05 compared to TM/NC group; n.s. indicates no signifcant difference). Data are presented as mean ± SEM. **B**, **C** and **D**, 2-way ANOVA followed by Bonferroni post-test or Tukey post-test. **E** and **F**, unpaired two-tailed Student’s *t*-test

We further investigated whether QRICH1 could regulate the progression of cardiac hypertrophy in cardiomyocytes. Treatment of NRCMs with angiotensin II (Ang II), phenylephrine (PE), or ISO induces cardiomyocyte hypertrophy. Our results confirmed the upregulation of QRICH1 in NRCMs under these hypertrophic stimuli (Supplementary Fig. 5A, B). NRCMs were transfected with an adenovirus encoding QRICH1 shRNA (AdshQRICH1) to induce QRICH1 knockdown (KD), followed by treatment with ISO (Supplementary Fig. 5C). Our data showed that knockdown of QRICH1 alleviated the hypertrophic response induced by ISO, as evidenced by a decrease in cell size and downregulation of fetal genes (Fig. 3A-D). Furthermore, QRICH1 KD in cardiomyocytes alleviated cell apoptosis following hypertrophic stimulation, which was consistent with apoptosis induced by tunicamycin (TM)-stimulated ER stress compared to the control (Fig. 3E). In addition, since QRICH1 is a transcriptional regulator of ER stress, we measured protein synthesis rates using puromycin labeling in non-targeting control (NC) and KD cardiomyocytes to monitor the requirement of QRICH1 for sustained translation regulation during ER stress. KD cardiomyocytes exhibited lower protein synthesis rates during late-stage ER stress induced by ISO, consistent with TM stimulation (Fig. 3F).

In contrast, cardiomyocytes infected with AdQRICH1 to overexpress QRICH1 (OE) (Supplementary Fig. 6A) amplified the effects of ISO on cardiomyocyte enlargement (Supplementary Fig. 6B-D) and the expression of hypertrophy markers ANP, BNP, and Myh7 (Supplementary Fig. 6E). Additionally, OE cardiomyocytes increased apoptosis in both TM and ISO stimuli (Supplementary Fig. 6F). Thus, these results indicate that downregulation of QRICH1 alleviates cardiomyocyte hypertrophy, whereas upregulation of QRICH1 exacerbates ISO-induced cardiac hypertrophy.

### QRICH1 interacts with ATF6 in cardiac muscle cells under stress

**Fig. 4:**
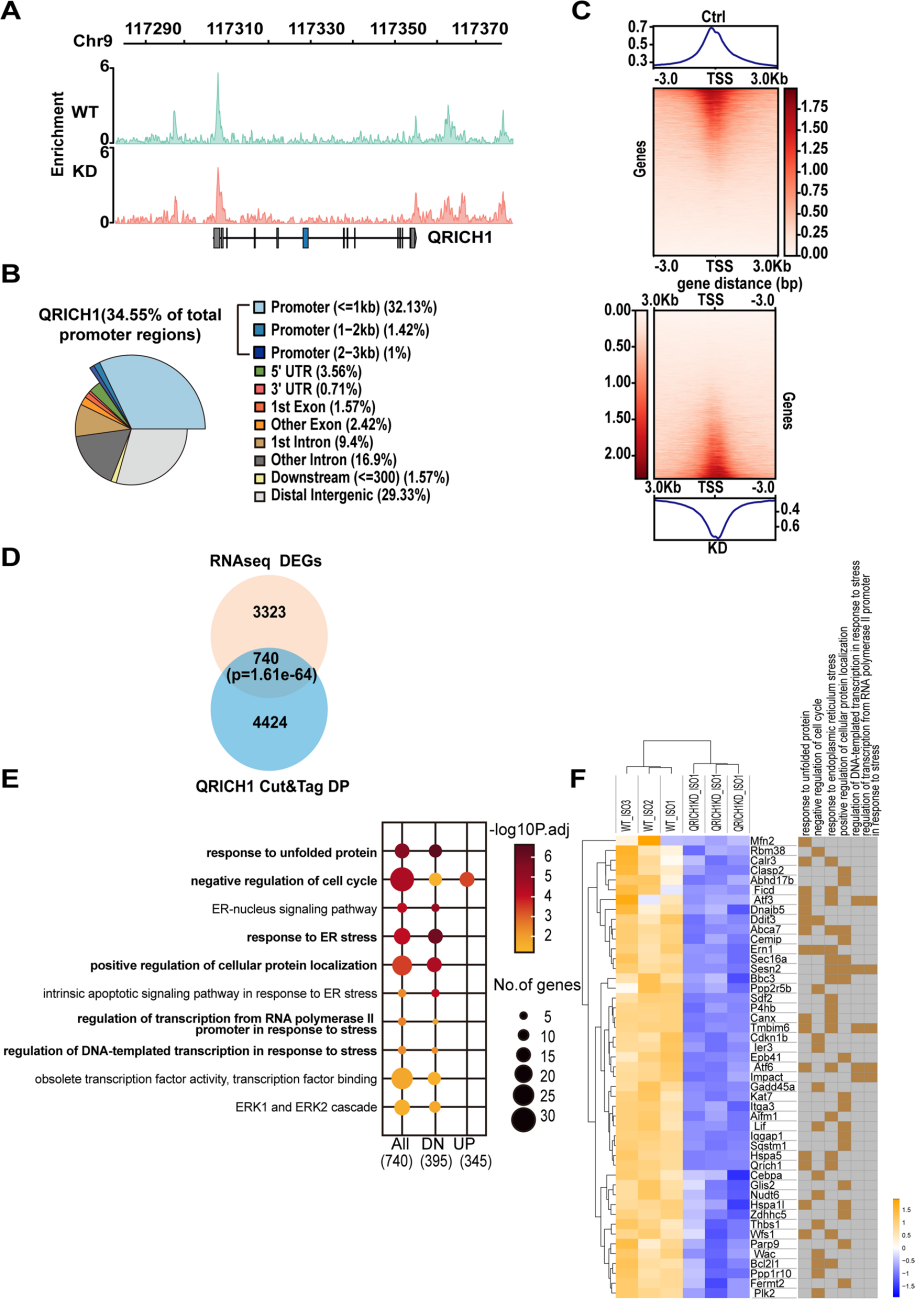
ATF6 is a target gene of QRICH1 under growth stimulation. **A**, QRICH1 binding profile of the QRICH1 promoter region in Ctrl and QRICH1 knockdown (KD) cardiomyocytes. **B**, Genomic annotation of QRICH1 Cut&Tag peak. The promoter regions are defined as the indicated distance from the TSS. **C**, Peak distribution of QRICH1 Cut&Tag within 3 kb from TSS. The heat map shows the read density of QRICH1 Cut&Tag. **D**, The Venn diagram shows the overlap of QRICH1 target genes in Cut&Tag and DEGs in Ctrl and QRICH1 KD cardiomyocytes responding to ISO treatment. **E**, Functional enrichment analysis of 740 overlapping gene sets in (D). All, 740 DEGs; DN 395 down-regulated genes in QRICH1 KD cells; UP 345 up-regulated genes in QRICH1 KD cells. **F**, The RNA-seq of Ctrl and QRICH1 KD cardiomyocytes was performed to show the response to ISO treatment. The heatmap displayed selective DEGs associated with specific biological processes related to (**E**). Yellow indicates the biological process involved by that gene

Next, we attempt to investigate the underlying mechanisms by which QRICH1 promotes cardiac damage. Since QRICH1 possesses a DUF3504 domain that shares homology with transposase-like DNA-binding proteins, we hypothesized that QRICH1 might affect the hypertrophy process by binding to DNA that regulates cardiomyocyte hypertrophy (Kojima and Jurka 2011). We performed sequencing of enzyme-tagged genomic DNA from wild-type (WT) or QRICH1 KD NRCMs after ISO treatment (Fig. 4A). We identified a total of 5358 QRICH1 binding peaks in the genome, with 34.55% of the QRICH1 peaks located within the gene promoter regions (Fig. 4B). Specifically, QRICH1 was positioned within the promoter regions of 1784 genes (Fig. 4B, C). Gene Ontology (GO) analysis of CUT&TAG data revealed that in the KD samples, QRICH1 preferentially binds to promoters of genes involved in RNA transport, nuclear transport, and protein localization to the nucleus. These promoters are located in regions with significantly increased chromatin accessibility compared to the WT (Supplementary Fig. 7A). Conversely, in KD samples where chromatin accessibility at promoters is significantly reduced, QRICH1 shows a preference for binding to promoters of genes associated with DNA metabolism and chromatin binding (Supplementary Fig. 7B). This observation suggests that QRICH1 maintains consistency in its structural and functional roles.

To identify potential target promoters that could influence cardiac hypertrophy, we compared the transcriptomes from WT versus QRICH1 KD cells after ISO treatment and identified 4063 differentially expressed genes (DEGs), with 1862 increased and 2201 decreased genes in KD cells (Fig. 4D, Supplementary Table 8). Eighteen percent of the upregulated genes and 18.5% of the downregulated genes were found to be potential targets of QRICH1 binding (Supplementary Table 8). The most enriched annotations for target genes activated by QRICH1 were “response to ER stress,” “transcription in response to stress,” and “proliferation,” while cell cycle genes are enriched among target genes suppressed by QRICH1 (Fig. 4E). Because maintaining normal gene transcription and protein translation under ER stress is crucial for preserving normal cellular function, it is noteworthy that in KD cells, the number of genes belonging to the pathways “regulation of DNA-templated transcription in response to stress” and “regulation of transcription from RNA polymerase II promoter in response to stress” decreased. Combined with RNA-seq analysis, ATF6, Tmbim6, ATF3, and Sesn2 are among the commonly downregulated genes (Fig. 4F). As a transcription factor in ER stress, ATF6 has been reported to be involved in the regulation of cardiac hypertrophy (Blackwood et al. 2019a). Accordingly, we speculated that QRICH1 influences the occurrence and development of cardiac hypertrophy by regulating ATF6.

**Fig. 5:**
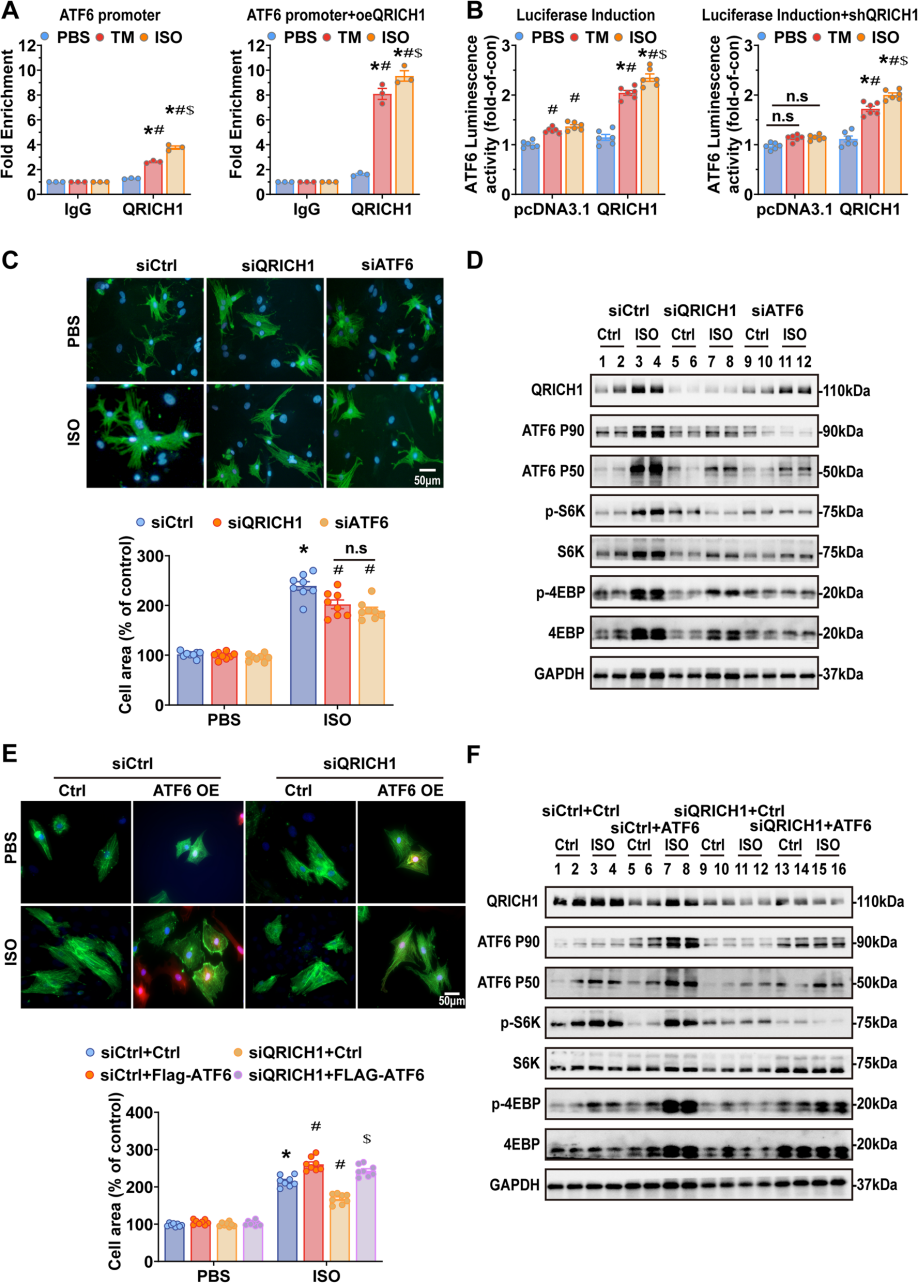
QRICH1 affects cardiac hypertrophy through the ATF6-mTOR pathway. **A**, Chromatin Immunoprecipitation (ChIP) analysis using a QRICH1-specific antibody to detect QRICH1 occupancy at the ATF6 promoter in NRCMs transfected with Adv.QRICH1 or Adv.NC, following treated with PBS, ISO and Tm for 48 h (*n* = 3 independent experiments, *P < 0.05 compared to IgG/PBS group, ^#^*P* < 0.05 compared to QRICH1/PBS group, ^$^*P* < 0.05 compared to QRICH1/TM group). **B**, Luciferase activity of the H9C2 cell extracts. ShQRICH1 or shQRICH1-NC cells were transfected with pcDNA3.1 or QRICH1 full-length plasmid and atf6 promoter luciferase reporter plasmid, followed by ISO treatment for each group of cells for 48 h (*n* = 6 independent experiments, *P < 0.05 compared to pcDNA3.1/PBS group, ^#^*P* < 0.05 compared to QRICH1/PBS group, ^$^*P* < 0.05 compared to QRICH1/TM group). **C**, Representative immunofluorescence images (upper) of α-actinin staining (green) and DAPI (blue) in NRCMs infected with siCtrl or siQRICH1 or siATF6 and treated with PBS or ISO for 48 h. Measurement (lower) of cell surface area after ICF (*n* > 100 cells per group, scale bars = 50 μm, *P < 0.05 compared to siCtrl/PBS group, ^#^*P* < 0.05 compared to siCtrl/ISO group, n.s. indicates no signifcant difference). **D**, Western blots of NRCMs infected with siCtrl or siQRICH1 or siATF6 and treated with PBS or ISO for 48 h. **E**, Representative immunofluorescence images (upper) of FLAG-ATF6 (red), α-actinin (green), DAPI (blue) in NRCMs infected with a control plasmid or a plasmid encoding Flag-ATF6 and either siCtrl or siQRICH1, followed by treatment ± ISO for 48 h. Measurement (lower) of cell surface area after ICF, FLAG-positive cells were used for cell surface area analysis (*n* > 100 cells per group, scale bars = 50 μm, *P < 0.05 compared to siCtrl + Ctrl/PBS group, ^#^*P* < 0.05 compared to siCtrl + Ctrl/ISO group, ^$^*P* < 0.05 compared to siQRICH1 + Flag-ATF6/ISO group). **F**, Western blots of NRCMs infected with a control plasmid or a plasmid encoding Flag-ATF6 and either siCtrl or siQRICH1, followed by treatment ± ISO for 48 h. Data are presented as mean ± SEM. 2-way ANOVA followed by Bonferroni post-test or Tukey post-test

We next determined the effect of QRICH1 on the transcriptional activity of ATF6. The ChIP-qPCR results showed that in cardiomyocytes overexpressing QRICH1 and treated with Tm and ISO, the enrichment of QRICH1 at the ATF6 promoter region increased, with ISO-induced QRICH1 enrichment being greater than that induced by Tm (Fig. 5A). Luciferase reporter gene analysis further indicated that knockdown of QRICH1 can inhibit the transcription of ATF6, while ectopic expression of QRICH1 increases the transcription of ATF6. However, QRICH1-mediated transcription of ATF6 requires stress stimulation (Fig. 5B). Altogether, these data show that QRICH1 influences the induction of ATF6 under cardiac stress.

### QRICH1 influences cardiac hypertrophy through the ATF6-mTOR pathway

To investigate the mechanism of interaction between QRICH1 and ATF6, we utilized loss-of-function strategies to explore the signaling pathways involved in ISO-induced hypertrophy in NRCMs. Knock down either QRICH1 or ATF6 mitigated the impact of ISO on cardiomyocyte hypertrophy, decreased the induction of fetal genes, and diminished the activation of the mTOR pathway (Fig. 5C, D; Supplementary Fig. 8A). To further validate the results obtained with ATF6 siRNA, we employed a different ATF6 loss-of-function involving the ATF6 inhibitor, Ceapin-A7 (Torres et al. 2019). In line with this result, Ceapin-A7 alleviated ISO-induced cardiomyocyte hypertrophy (Supplementary Fig. 8B).

To complement the loss-of-function approach for QRICH1, we employed a gain-of-function approach to investigate the effects of ectopic expression of QRICH1 and ATF6. As an inhibitor of mTORC1, Rapamycin can block the cell hypertrophy promoted by ISO. Overexpression of QRICH1 exacerbates ISO-induced cardiomyocyte hypertrophy, and rapamycin also blocks this effect (Supplementary Fig. 8C). Moreover, the overexpression of QRICH1 was not able to restore growth in ISO-induced cells treated with ATF6 siRNA or Ceapin-A7 (Supplementary Fig. 8B). Consistent with our expectations, in the absence of ISO stimulation, overexpression of ATF6 has no effect on cell hypertrophy; however, under growth stimulation, overexpressed ATF6 can completely restore the loss of cell area caused by QRICH1 knockdown and reactivate mTORC1 (Fig. 5E, F; Supplementary Fig. 8D). Furthermore, overexpression of QRICH1 did not reverse the improvement in cardiomyocyte hypertrophy observed following ATF6 knockdown under ISO stimulation (Supplementary Fig. 8E). Meanwhile, knockdown of QRICH1 suppressed ISO-induced expression of BIP, CHOP, and XBP1 in cardiomyocytes, and overexpression of ATF6 significantly reversed this suppression (Supplementary Fig. 8F). Interestingly, neither QRICH1 knockdown nor overexpression significantly altered Atf6β mRNA levels or protein expression in NRCMs (Supplementary Fig. 8G, H). Taken together, our results demonstrate that QRICH1 affects the activation of the mTOR signaling pathway by regulating ATF6.

### Overexpression ATF6 restored the cardiac growth in QRICH1 knockdown mice subjected to ISO

**Fig. 6:**
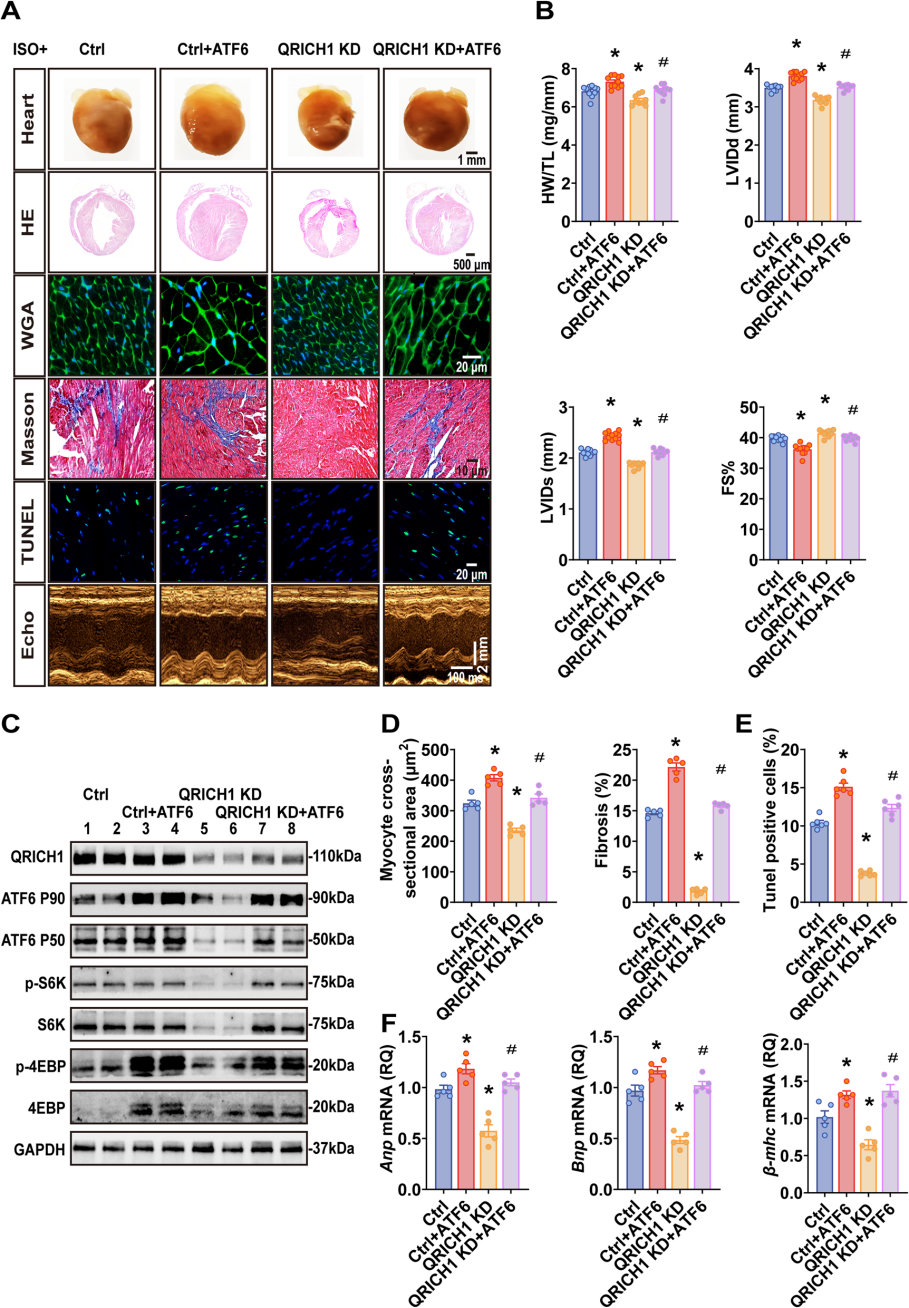
Effect of cardiac myocyte-specific ectopic ATF6 expression in QRICH1 knockdown mice hearts treated with ISO. **A**, Representative gross morphology of mouse hearts (the top row, scale bars = 1 mm), cross-sections of the heart stained with Hematoxylin and Eosin (the second row, scale bars = 500 μm), cell boundaries stained with wheat germ agglutinin (the third row, scale bars = 20 μm), LV fibrosis stained with Masson's trichrome (the forth row, scale bars = 10 μm), LV apoptotic cell stained with TUNEL (the fifth row, scale bars = 20 μm), M-mode echocardiography images of the LV chamber in Ctrl/Ctrl + ATF6/QRICH1 KD/QRICH1 KD + ATF6 mice 4 weeks after ISO injection (the bottom row, scale bars = 2 mm). **B**, The ratio of heart weight to tibia length (HW/TL) and echocardiographic measurements of LV end-diastolic internal diameter (LVIDd), LV end-systolic internal diameter (LVIDs), and fractional shortening (FS) in Ctrl/Ctrl + ATF6/QRICH1 KD/QRICH1 KD + ATF6 mice 4 weeks after ISO injection (*n* = 10, 11, 9, 10 mice from left to right). **C**, Western blots of LV in Ctrl/Ctrl + ATF6/QRICH1 KD/QRICH1 KD + ATF6 mice 4 weeks after ISO injection. **D**, Statistical results for quantification of cell cross-sectional area and myocardial interstitial collagen (*n* = 5 mice per group). **E**, Statistical results for quantification of cell apoptosis (*n* = 6 mice per group). **F**, Measurement levels of myocardial hypertrophy-associated transcripts ANP (atrial natriuretic peptide), BNP (brain natriuretic peptide), β-MHC (β myosin heavy chain) (*n* = 5 mice per group). *P < 0.05 compared to Ctrl group. ^#^*P* < 0.05 compared to QRICH1 KD group. Data are presented as mean ± SEM. unpaired two-tailed Student’s *t*-test

To confirm that the effect of QRICH1 promoting cardiac hypertrophy in vivo is due to the activation of ATF6 in cardiomyocytes under growth stimuli, we used dual AAV9 injections to evaluate the effects of ATF6 overexpression on cardiac phenotypes in QRICH1 KD mice (Supplementary Fig. 9A-C). In response to ISO, in QRICH1 KD mice, overexpression of ATF6 reactivated the mTOR signaling pathway, resulting in exacerbated cardiac hypertrophy and dysfunction (Fig. 6A-C; Supplementary Fig. 9D, E; Supplementary Table 5). This effect was further evidenced by increased myocardial fibrosis, elevated cellular apoptosis, enhanced inflammatory responses, and upregulation of fetal gene expression (Fig. 6D-F; Supplementary Fig. 9F). Moreover, both mRNA and protein levels of ATF6 were indeed significantly increased in human LVH samples compared to controls (Supplementary Fig. 9G). Taken together, these data suggest that QRICH1 exacerbates stress-induced pathological cardiac hypertrophy, potentially through the activation of the mTOR signaling pathway and its interaction with ATF6.

## Discussion

ER stress exhibits a strong association with risk factors that contribute to cardiovascular disease (CVD), such as inflammation, apoptosis, oxidative stress, and dysregulation of autophagy (Ren et al. 2021; Lemmer et al. 2021). These factors are implicated in both physiological and pathological conditions within the cardiovascular system. Notable examples of such conditions include hypoxia (oxygen deprivation) and fuel deprivation, pressure overload, dilated cardiomyopathy, hypertrophy, and heart failure (Liu et al. 2024; Lu et al. 2020; Xu et al. 2024). In pressure overload-induced mouse models, certain ER stress genes showed elevated expression, suggesting ER protein misfolding (Hu et al. 2020). As a major transducer of ER stress, PERK is required to protect the heart from pressure overload-induced congestive heart failure (Liu et al. 2014). Existing research has confirmed that QRICH1, as an arm under the PERK-eiF2α pathway, influences the outcomes of endoplasmic reticulum stress by controlling protein homeostasis through transcriptional regulation (You et al. 2021). However, the activation and functions of QRICH1 in cardiac myocytes during hemodynamic overload remain unexplored. Our current findings demonstrate that QRICH1 may act as a significant regulator in managing pathological cardiac hypertrophy (Supplementary Fig. 10).

QRICH1 is a conserved gene ubiquitously expressed in mammals (Kumble et al. 2022). Variants in QRICH1 have been associated with developmental delay, neurodevelopmental disorders, renal agenesis, and congenital heart disease (Baruch et al. 2021; San Agustin et al. 2016; Li et al. 2015), underscoring its importance in maintaining the normal structure of tissues with high protein synthesis demands. Cardiac hypertrophy increases the requirement for protein folding, leading to an accumulation of misfolded proteins in the ER (Blackwood et al. 2023). In this study, continuous pressure overload and hypertrophic stimuli led to cardiac dysfunction, an increase in cardiac ANP secretion, and the activation of QRICH1. The activation of QRICH1 may lead to an elevated synthesis rate of terminal ER stress proteins, resulting in the accumulation of proteotoxicity (You et al. 2021). However, the expression of ANP is attenuated in cardiomyocytes with QRICH1 knockdown, consistent with the specific reduction of protein flux into the ER observed upon QRICH1 inhibition. Although the precise timing of QRICH1 activation in pathological cardiac hypertrophy remains elusive, the concurrent increase of ANP suggests that the activation of cardiac myocyte QRICH1 is dependent on the stress conditions of heart failure.

In addition to its role in maintaining cellular protein homeostasis, QRICH1 plays a critical role under pathological conditions such as inflammatory or metabolic diseases, suggesting its involvement in various cardiovascular diseases, including pathological cardiac hypertrophy. ER stress has been observed in pressure-induced cardiac hypertrophy and heart failure (Liu et al. 2014). Previous studies have shown that QRICH1 is activated by pathological ER stress in the mouse intestinal epithelium (You et al. 2021); however, its role in LVH under pathological ER stress has not been previously identified. LVH is a common condition in patients with hypertension, which can lead to abnormal cardiac function and stiffness (London 2003). Continuous overload can result in maladaptive hypertrophy, further progressing to overload cardiomyopathy and heart failure (Heimark et al. 2023). We observed an elevation of QRICH1 in LVH in both human and rodent models, with its localization in the cell nucleus closely associated with transcription and translation. Similarly, in QRICH1 knockdown mice subjected to hypertrophic stimuli, the silencing of QRICH1 alleviated interstitial fibrosis associated with LVH.

Chronic inflammation is considered a hallmark of heart dysfunction (Harding et al. 2023; Schiattarella et al. 2021; Sopic et al. 2023). When endoplasmic reticulum stress is prolonged or excessive, apoptosis signals mediated by the ER are triggered, including the upregulation of C/EBP homologous protein (CHOP) in the heart following TAC, indicating that ER-initiated apoptosis is intensified under these conditions (Fu et al. 2010). Intestinal epithelial cells with QRICH1 deficiency demonstrate increased resilience to apoptosis mediated by ER stress (You et al. 2021). Thus, QRICH1 is also implicated in mediating inflammatory signals and programmed cell death. Our findings align with the role of QRICH1 in inflammation. In the TAC-induced model, there was a significant reduction in TNF-α, IL-6, and IL-1β, accompanied by a noticeable alleviation of fibrosis when QRICH1 was present. Additionally, QRICH1 knockdown in cardiac tissue led to reduced apoptosis and promoted cell survival. Tachyarrhythmia such as atrial fibrillation and ventricular tachycardia can also trigger chronic inflammation, impacting heart function (Hadaya et al. 2023; Pavlicek et al. 2019). Furthermore, ISO, which simulates catecholamine activation of cardiac β-receptors, induces tachycardia (Wallner et al. 2016; Hayashi et al. 2023). In mice induced with ISO, we observed that QRICH1 knockdown showed consistent trends in inflammation markers, fibrosis, and apoptosis, as seen in the TAC model. These findings suggest that the expression of QRICH1 in cardiac myocytes mediates maladaptive responses in pathological cardiac hypertrophy.

ATF6 is a critical component of the unfolded protein response (UPR), which is essential for maintaining the functionality of the endoplasmic reticulum (ER) as cells adapt to stress (Fu et al. 2008). In the heart, ATF6 responds to various stimuli by activating not only traditional ER stress-response genes but also a range of non-canonical genes. This dual role highlights its crucial function in managing cellular stress and preserving ER integrity (Glembotski et al. 2020).This diversity extends to the induction of specific ATF6-dependent genes in response to different cardiac stimuli. The activation of ATF6 in ischemia/reperfusion (I/R) injury, as demonstrated in wild-type mice, suggests that ATF6-induced genes are critical in protecting against I/R damage and preserving cardiac function (Blackwood et al. 2019b). However, activated ATF6 exhibits non-canonical roles in growth-driven during pressure overload-induced pathological cardiac hypertrophy through the ATF6-Rheb-mTORC1 axis (Blackwood et al. 2019a). mTORC1 is one of the complexes in the mTOR pathway. mTORC1 plays a crucial role in orchestrating a broad spectrum of biological functions, such as growth, metabolism, protein synthesis, and autophagy. S6K and 4EBP1 are key downstream effectors in the mTORC1 signaling pathway, mediating its regulatory effects on these processes (Saxton and Sabatini 2017; Sciarretta et al. 2018). Furthermore, inhibiting the mTOR signaling pathway can mitigate myocardial hypertrophy induced by pressure overload, suggesting a protective role (Shioi et al. 2003; McMullen et al. 2004). Rheb, as an activator of the mammalian target of mTORC1, has been demonstrated to act as an interrupter by activating PERK and phosphorylating eIF2α to inhibit protein synthesis (Tyagi et al. 2015). This, in turn, proves the connection between the ATF6 and PERK pathways from another perspective.

QRICH1 functions as a distinct arm of the PERK-eIF2α pathway and operates in parallel to ATF4 (You et al. 2021). It is predominantly located in the nucleus under both ER stress and normal conditions, where it regulates a transcriptional network that oversees protein translation and secretory processes. This role underscores its significance in modulating cellular responses to maintain homeostasis and adapt to stress within the endoplasmic reticulum environment (You et al. 2021). Our RNA-seq and Cut&Tag analyses have revealed that QRICH1 collaborates with ATF6 in maintaining ER proteostasis. Utilizing luciferase reporter assays and ChIP-qPCR, we identified that QRICH1 regulates the transcription of ATF6 in response to growth stimuli. Furthermore, gene silencing of ATF6 confirmed its role in attenuating cardiac myocyte hypertrophy, which was further validated by the observed reduction in the area of isoproterenol (ISO)-induced cardiomyocytes treated with the ATF6 inhibitor Ceapin (Xue et al. 2021). By inhibiting the cleavage of ATF6α, Ceapin-A7 effectively blocks ATF6α signaling without affecting its expression or activation in response to ER stress. It is important to note that in this study, the regulation of ATF6 transcription by QRICH1 is dependent on growth stimuli. Hence, the beneficial effects of QRICH1 suppression on cardiac hypertrophy were reversed by ATF6 overexpression under growth stimuli. Our findings suggest that the QRICH1-ATF6 interaction modulates mTOR activation, offering new insights into the mechanisms underlying cardiac stress responses.QRICH1 knockout does not significantly affect ER homeostasis or cell survival under non-stress conditions but induces resistance to UPR-mediated cell death under stress (You et al. 2021). This aligns with our observations in this study, where QRICH1 knockdown did not impact cardiomyocyte growth and apoptosis under non-stress conditions. Given that pathological myocardial hypertrophy is often diagnosed alongside clinical symptoms, spatiotemporal targeted interventions are crucial (Cui et al. 2021). In our study, we utilized tissue-specific AAV9 virus transfection to knock down QRICH1 in cardiomyocytes, which delayed myocardial fibrosis, reduced apoptosis, and improved myocardial remodeling in a mouse model of pathological hypertrophy. This approach suggests a potential therapeutic option for patients with primary or secondary hypertrophic cardiomyopathy, particularly those who have not undergone surgery or are considering it. Looking forward, advancements in CRISPR technology with spatiotemporal editing capabilities, as well as high-throughput screening of chemical libraries to identify small molecule inhibitors that specifically bind and inhibit QRICH1 activity, present promising avenues for targeting QRICH1 in the treatment of myocardial hypertrophy (Akoumianakis et al. 2022; Jackson et al. 2022; Reid et al. 2016).

### Limitations

This study has certain limitations. Firstly, while QRICH1 is proposed to interact with ATF6 as a transcriptional regulator, it may interact with other stress-responsive pathways such as NF-κB-mediated inflammation, JNK/p38 MAPK signaling, or oxidative stress pathways, all of which have been implicated in cardiac hypertrophy and remodeling. Furthermore, given the findings from prior studies showing enhanced decompensation in long-term pressure overload conditions in both Atf6 and Atf6β null mice (Correll et al. 2019), investigating the regulation of ATF6 transcription by QRICH1 under chronic hemodynamic stress becomes crucial. This approach aims to elucidate QRICH1's role at various stages from compensation to decompensation and potentially heart failure, thus providing insights into therapeutic interventions. Secondly, although AAV9-mediated gene manipulation is a powerful tool for dissecting gene function, the long-term effects and potential off-target effects of this approach in the context of cardiac function and hypertrophy remain to be fully elucidated. Third, although we demonstrated increased QRICH1 expression in human LVH samples, further validation using human primary cardiomyocytes, induced pluripotent stem cell (iPSC)-derived cardiomyocytes, or relevant human cardiac tissues is crucial to enhance the translational relevance of our findings. Lastly, our study primarily addresses maladaptive hypertrophy, and it remains unclear whether QRICH1 also plays a role in adaptive hypertrophy. Further research is required to clarify this aspect of QRICH1 biology.

## Conclusion

In conclusion, our study demonstrates that QRICH1 affects the progression of pathological cardiac hypertrophy by regulating the transcription of ATF6. Knockdown of QRICH1 reduces apoptosis and inflammation during hypertrophy. Then, QRICH1 may be a new diagnostic and therapeutic approach for pathological cardiac hypertrophy and hypertrophy-induced heart failure.

## Supplementary Information

Supplementary Material 1.

Supplementary Material 2.

## Data Availability

The de novo assembled genomes and raw sequencing reads have been deposited in the NCBI Sequence Read Archive (SRA) under the BioProject accession number PRJNA1226899. The corresponding SRA accession number is SRR32484789, SRR32484788, SRR32489841 and SRR32489840.

## Abbreviations

QRICH1: Glutamine-rich protein 1
LVH: Left ventricular hypertrophy
NRCMs: Neonatal rat cardiomyocytes
TAC: Transverse aortic constriction
ISO: Isoproterenol
Tm: Tunicamycin
Rap: Rapamycin
PBS: Phosphate buffered saline
AAV: Adeno-associated virus
AdV: Adenovirus
eIF2α: Eukaryotic Initiation Factor 2α
ATF6: Activating transcription factor 6
mTOR: Mammalian target of rapamycin
ANP: Atrial natriuretic peptide
BNP: Brain natriuretic peptide
β-MHC: β Myosin heavy chain
ER: Endoplasmic reticulum
Cut & Tag: Cleavage under targets and tagmentation
RNA-seq: RNA sequencing
DEGs: Differentially expressed genes
DP: Differentially peak
TSS: Transcription initiation site
KD: Knockdown
OE: Overexpression
