# Supplementary material for: QRICH1 regulates ATF6 transcription to affect pathological cardiac hypertrophy progression
Source: Mol Med. 2025 May 13;31:183. doi: 10.1186/s10020-025-01241-2 (PMC12070701; doi:10.1186/s10020-025-01241-2)
Supplement: Supplementary file 1 — Supplementary Material 1. [file 10020_2025_1241_MOESM1_ESM.docx]

**SUPPLEMENTAL MATERIALS**

**QRICH1 regulates ATF6 transcription to affect pathological cardiac hypertrophy progression**

**Short title:** QRICH1 exacerbates pathological hypertrophy

Lihui Zhang^1,2^, Hongping Chen^4^, Guangmei Zou^3^, Wenjuan Jia^2^, Haibin Dong^2^, Chunxiao Wang^2^, Hua Wang^2^, Yugang Liu^2^, Da Teng^1,2^, Bowen Xu^1,2^, Lin Zhong^2^*, Lei Gong^2^*, Jun Yang^2^*

^1^Medical college, Qingdao University, Qingdao, Shandong, China

^2^Department of Cardiology, Affiliated Yantai Yuhuangding Hospital of Qingdao University, Yantai, Shandong, China

^3^Department of Cardiac Surgery, Affiliated Yantai Yuhuangding Hospital of Qingdao University, Yantai, Shandong, China

^4^Department of Cardiology, The Affiliated Hospital of Xuzhou Medical University, Xuzhou, China

***Correspondence to:**

Lin Zhong, Lei Gong and Jun Yang

Department of Cardiology

Affiliated Yantai Yuhuangding Hospital of Qingdao University

20 Yudong Road, Zhifu District, Yantai, Shandong 264000, China

Tel: 86-0535-6691999

E-mail: [zhonglinyhd@qdu.edu.cn](mailto:zhonglinyhd@qdu.edu.cn), [leigong27@163.com](mailto:leigong27@163.com), [yangjyhd@qdu.edu.cn](mailto:yangjyhd@qdu.edu.cn)

Lihui Zhang and Hongping Chen have contributed equally to this work.

**Keywords** Cardiac remodeling, Heart failure, Endoplasmic reticulum stress, Pressure overload

**Expanded Methods and Materials**

**Animals**

Male C57BL/6J mice were bred and housed at the Experimental Animal Center of The Fourth School of Clinical Medicine, Qingdao University, Yantai, China, and were obtained from Jinan Pengyue Laboratory Animal Breeding Co., Ltd. Throughout the study, mice were maintained under optimal living conditions, including a stable temperature of 22°C and a consistent 12-hour light-dark cycle. They had unrestricted access to water and food. For experimental purposes, mice were randomly assigned to their respective groups and uniquely identified for tracking. Furthermore, to ensure the integrity of the experimental outcomes, the researchers conducting the assessments were blinded to the group allocations of the individual mice. For anesthesia in mice, isoflurane was typically used at concentrations of 3% to 5% mixed with oxygen for induction and reduced to 1.5% to 2.5% for maintenance, delivered through an inhalation chamber or nose cone. For euthanasia, mice were anesthetized via intraperitoneal injection with pentobarbital sodium (60 mg/kg, Sigma-Aldrich). After they lost the pedal withdrawal reflex, euthanasia was then performed through cervical dislocation. This study's animal protocols were rigorously reviewed and received approval from the Institutional Animal Care and Use Committee of Qingdao University Medical College and the Ethics Committee of Yantai Yuhuangding Hospital (Approval No. 2023-329), ensuring compliance with the National Institutes of Health's Guidelines for the Care and Use of Laboratory Animals.

**Human heart tissues**

The research entailed the analysis of two categorically distinct human heart tissue types: samples from individuals diagnosed with left ventricular hypertrophy (LVH) (n=4) and samples from healthy controls (n=4). These specimens were precisely harvested from the myocardial segment of the left ventricular outflow tract (LVOT). Tissues exhibiting hypertrophy were collected from patients subjected to LVOT myotomy-myectomy procedures to address LVH. The regimen for managing heart failure in these patients included the administration of β-blockers and diuretics. The healthy human heart tissues were obtained from potential multi-organ donors who had a normal circulatory supply but succumbed to head trauma or intracranial hemorrhage, rendering their hearts unsuitable for transplantation due to technical limitations or non-cardiac-related issues. Immediately after collection, the tissues were quickly immersed in liquid nitrogen for preservation and then stored at -80°C for future analysis. This study was sanctioned by the Ethics Committee of Yantai Yuhuangding Hospital (Approval No. 2023-328), in strict compliance with the ethical standards stipulated in the 1975 Declaration of Helsinki. The STROBE checklist used in this sub-study was provided in the Supplementary Material. Informed consent was duly obtained from all participants prior to their involvement in the study.

**Gene Expression Modulation Using Adeno-Associated Virus**

Using recombinant adeno-associated virus serotype 9 (rAAV9) obtained from OBioTechnology and Genechem, we manipulated the expression levels of QRICH1 and ATF6 in vivo. Male C57BL/6J mice, aged 3-4 weeks, were randomly assigned to receive a single-tail vein injection. Each mouse was administered 100μl containing 5×10^11^ genome copies/mL of one of the following constructs: for knockdown, pAAV-cTNT-GdGreen-miR30shQRICH1-WPRE or pAAV-cTNT-GdGreen-miR30shNC-WPRE; for overexpression, pAAV-cTNT-QRICH1-3Flag-T2A-EGFP, pAAV-cTNT-3Flag-T2A-EGFP, pAAV-cTNT-Atf6-mCherry-3xFLAG-WPRE, or pAAV-cTNT-mCherry-3xFLAG-WPRE. Five weeks post-injection, the efficacy of QRICH1 and ATF6 knockdown or overexpression was evaluated using Western blot analysis and immunofluorescence techniques. The shRNA sequence designed to target mouse QRICH1 was 5’-GGAGCATATCCCACATCAACA-3’.

**Construction of lentivirus**

Within a BSL-2 facility, shQRICH1 H9C2 stable cells or shQRICH1-NC were produced by transfecting 293T cells with pmiRZip-shQRICH1 or pmiRZip-shQRICH1-NC and packaging plasmids (pMDLg/pRRE, pRSV-Rev, pMD2.G) using a 3:1:1:1 ratio via calcium phosphate or PEI. After 48 and 72 hours, lentiviral particles were harvested, filtered (0.45 μm), and optionally concentrated. H9C2 cells were transduced with optimized lentivirus, followed by puromycin selection to establish stable lines. QRICH1 knockdown is confirmed by qRT-PCR or Western blot. Viruses were stored at -80°C, ensuring adherence to biosafety protocols. One day after plating, cardiomyocytes were co-incubated with recombinant adenovirus for 2 hours. Following the removal of the viral suspension, the cells were placed in maintenance medium for one day before being subjected to stimulation with various drugs. The multiplicity of infection (MOI) of the virus was 10.

**Animal models**

In this study, male C57BL/6J mice, aged 8 weeks and weighing between 23-25 g, were utilized to investigate the effects of aortic ligation on cardiac function. Initially, the mice were anesthetized in an induction chamber using a 5% isoflurane/O_2_ mixture, followed by the maintenance of anesthesia via a nasal cone delivering 1.5-2.5% isoflurane, complemented by endotracheal intubation to ensure adequate ventilation during surgery. To manage pain, 5 mg/kg of carprofen was administered subcutaneously 30 minutes before the procedure and subsequently once every 24 hours for three days post-surgery. The depth of anesthesia was meticulously assessed by monitoring breathing frequency, responsiveness to toe pinch, and the degree of muscular laxity. The surgical intervention involved a precise incision at the left second rib to perform aortic ligation, using a 6-0 silk suture and a 28G needle for the ligation, after which the chest cavity was immediately sutured to minimize the risk of complications. A sham-operated group was included, undergoing all procedural steps except the aortic ligation, serving as a control for the surgical intervention. Additionally, isoprenaline HCl (ISO, Glpbio Technology) was administered subcutaneously at a dose of 5 mg/kg in multiple injections twice daily to induce cardiac hypertrophy, while the control group received physiological saline.

**Echocardiography**

Four weeks following the transverse aortic constriction (TAC) procedure and isoprenaline (ISO) administration, the cardiac structure and function of the mice were evaluated via transthoracic echocardiography, employing the VINNO 6 LAB system. For the echocardiographic examination, mice were minimally anesthetized with 1% isoflurane, delivered at a flow rate of 1 L/min, and positioned on a heated pad subsequent to chest hair removal. Electrocardiogram leads were affixed to monitor cardiac activity, and a high-frequency 23 MHz transducer was utilized to capture comprehensive cardiac images. These images encompassed the parasternal long-axis and short-axis views at the papillary muscle level, alongside two-dimensional M-mode, color Doppler, tissue Doppler, and spectral Doppler imaging. Critical measurements were taken to assess left ventricular (LV) wall thickness, internal diameter, overall LV function, and carotid artery blood flow. The specific parameters measured included the interventricular septal thickness at end-diastole (IVSd) and end-systole (IVSs), LV internal diameter at end-diastole (LVIDd) and end-systole (LVIDs), LV posterior wall thickness at end-diastole (LVPWd) and end-systole (LVPWs), fractional shortening (FS), ejection fraction (EF), and the E-to-e’ ratio (E/e’). For accuracy, at least three cardiac cycles were recorded for each parameter, from which the average values were computed. To ensure the objectivity of the data analysis, it was conducted by experienced researchers who were not privy to the initial treatment groups of the mice.

**Histological analysis**

Subsequently, mouse cardiac tissues were processed, including fixation in 4% paraformaldehyde (Solarbio), dehydration, paraffin embedding, and sectioning at 4 μm for histological examination. Tissue sections underwent staining with hematoxylin-eosin for structural visualization, FITC-labeled wheat germ agglutinin (Sigma-Aldrich) for cardiomyocyte boundary delineation, and Masson’s trichrome (Solarbio) for fibrosis identification. Images of cardiomyocytes, captured at 160x magnification using an Axio Observer 7 microscope (Carl Zeiss), were quantitatively analyzed with ZEN 3.4 software (Carl Zeiss), tracing over 200 cells across multiple samples. Fibrosis quantification was conducted using ImageJ by comparing the area of fibrosis (blue-stained) to the total ventricular area in images from a DMLB2 microscope (Leica), facilitating a comprehensive analysis of cardiac tissue remodeling.

**Immunohistochemistry and Immunofluorescence staining**

For immunohistochemical staining, myocardial sections underwent dewaxing, antigen retrieval with Citrate Solution (Sangon Biotech), and blocking of endogenous peroxidase with 3% H_2_O_2_ and goat serum. Overnight incubation at 4°C with anti-QRICH1 antibody (HPA037677, 1:200, Sigma Aldrich) preceded HRP-conjugated secondary antibody application and DAB staining (ZSGB-BIO), followed by hematoxylin counterstaining and imaging on a DMLB2 microscope.

Immunofluorescence of cardiac tissue involved fixing in 4% paraformaldehyde, dehydration in sucrose, embedding in OCT, and sectioning. Sections were permeabilized with Triton X-100, blocked with goat serum, and incubated with primary antibodies against QRICH1 or α-actinin α2 (clone EA-53, 1:400, Sigma Aldrich). Alexa Fluor® conjugated secondary antibodies and DAPI counterstaining were used before imaging with an Axio Observer 7 (Carl Zeiss). Cardiomyocytes were fixed, permeabilized, blocked in goat serum, and incubated with primary and fluorescent secondary antibodies, with DAPI for nuclei. Imaging was conducted on an Axio Observer 7, and cell size analysis was performed with ImageJ 1.52i software.

**Cell culture**

Primary cardiomyocytes were isolated from 1- to 2-day-old Sprague-Dawley rats using enzymatic dissociation with trypsin and collagenase, followed by density gradient centrifugation using Percoll (GE Healthcare) for cell separation. The neonatal rat cardiomyocytes (NRCMs) were subsequently cultured in DMEM-F12 medium supplemented with 10% fetal bovine serum (FBS) and antibiotics (100 units/mL penicillin and 100 μg/mL streptomycin), with 0.1 mM 5-bromo-2’-deoxyuridine (BrdU) added on the first day to inhibit non-cardiomyocyte proliferation.

Adult mouse hearts, previously infected with AAV9, underwent Langendorff perfusion to facilitate enzymatic dissociation, with collagenase being the primary enzyme used. After dissociation, cardiomyocytes were cultured in DMEM-F12 medium supplemented with 10% FBS for nutrients, insulin-transferrin-selenium (ITS, 1:100, Sigma-Aldrich) for metabolism support, and antibiotics (100 units/mL penicillin, 100 μg/mL streptomycin) for contamination prevention.

The H9C2, HEK293, and HEK293T cell lines, obtained from ATCC, were cultured in DMEM supplemented with 10% FBS and antibiotics.

**Plasmid and siRNA**

pcADV-EF1-mScarlet-CMV-Qrich1-3xFLAG was generated by cloning the rat QRICH1 gene into pcADV-EF1-mScarlet-CMV-MCS-3xFLAG. pcDNA3.1-3×FLAG-QRICH1 was generated by cloning the human QRICH1 gene into pcDNA3.1-3×FLAG. pSLenti-CMV-Atf6-Linker-mCherry-3xFLAG-WPRE was generated by cloning the rat ATF6 gene into pSLenti-CMV-mCherry-3xFLAG-WPRE. QRICH1shRNA (shQRICH1-1/2/3) is generated by inserting shRNA sequences into the pADV-U6-shRNA-CMV-EGFP vector. The shRNA sequences for QRICH1 were as follows: rat-shQRICH1-1:5’-GGAGCATATCCCACATCAACA-3’; rat-shQRICH1-2:5’-GCAGAGCATTACCCACATTGC-3’; rat-shQRICH1-3:5’-GCTCAGACTGTACATATATGG-3’. The luciferase reporter plasmid driven by ATF6 binding sites (ATF6-LUC) was purchased from Sangon Biotech. The rat ATF6 siRNA and the scramble control were purchased from Sangon Biotech, and siRNA sequences were as follows: Atf6 siRNA-1: sense 5’-CCAGUUUGAUGCAGCACAUTT -3’, antisense 5’-AUGUGCUGCAUCAAACUGGTT-3’; Atf6 siRNA-2: sense 5’- GGAGACAGCAGCGUAUGAUTT-3’, antisense 5’-AUCAUACGCUGCUGUCUCCTT-3’; Atf6 siRNA-3: sense 5’-GCUGUCCAGUACACAGAAATT-3’, antisense 5’- UUUCUGUGUACUGGACAGCTT-3’; Control siRNA: sense 5’-TTCUCCGAACGUGUCACGUTT-3’, antisense 5’-ACGUGACACGUUCGGAGAATT-3’.

**Recombinant adenovirus infection**

Adenovirus vectors were generated using the AdMax system (Microbix, Canada), following the manufacturer's protocols. Shuttle vectors harboring the target gene were co-transfected with adenovirus backbone plasmids into HEK293 cells to produce recombinant adenoviruses. After transfection, the viruses underwent large-scale amplification in HEK293 cells and were subsequently purified. The viral titer was quantified, and the expression of the target gene was verified through appropriate assays prior to their application in subsequent experiments.

**Enzyme-linked immunosorbent assay (ELISA)**

Peripheral blood was collected from mice and subjected to centrifugation at 4000 rpm for 10 minutes to separate the serum from cellular components. The concentrations of pro-inflammatory cytokines, including interleukin 6 (IL-6), interleukin 1β (IL-1β), and tumor necrosis factor-alpha (TNF-α), in the serum were quantified using ELISA kits, following the protocols provided by the manufacturer (Jonln).

**FACS analysis for apoptosis**

After 48 hours of cell treatment, cells were digested with 0.05% trypsin without EDTA, washed, and subjected to dual staining with Annexin V/propidium iodide (PI) labeled with fluorescein isothiocyanate (FITC, Yeasen Biotechnology) or dual staining with Annexin V/7-aminoactinomycin D (7-AAD) labeled with allophycocyanin (APC, Elabscience). The stained cells were then analyzed using a Moflo XDP flow cytometer (Beckman). Cells negative for PI/7-AAD staining were identified as viable cells and subjected to further analysis. Cells positive for Annexin V and negative for PI/7-AAD staining were classified as apoptotic or early apoptotic cells. The data were analyzed using NovoExpress software (Agilent Technologies). The numerical values represent the average of three independent experiments.

**Metabolic labeling with puromycin (SUnSET assay)**

In this study, we employed the SUnSET technique for metabolic labeling with puromycin in cardiac myocytes, adhering to protocols previously established [1, 2]. Initially, cardiac myocytes were transfected with either shRNA targeting QRICH1 (shQRICH1) or a non-targeting control shRNA (shNC). Following transfection, cells were treated with ISO or tunicamycin (Tm) for predetermined periods. After treatment, myocytes were incubated in a labeling medium containing 10 mg/mL puromycin for 30 minutes to facilitate the incorporation of puromycin into nascent polypeptides. Cells were then lysed, and protein concentrations were quantified via the Bicinchoninic Acid (BCA) assay. Proteins were resolved by SDS-PAGE, and puromycin incorporation was detected on membranes using an anti-puromycin antibody (MABE343, Sigma) at a dilution of 1:1000. To ensure accurate quantification, signals were normalized to GAPDH, serving as a loading control.

**RNA-seq analysis**

Total RNA was extracted from the samples using the Trizol reagent, followed by an assessment of RNA quality to ensure suitability for sequencing. RNA libraries were then prepared using the TruSeq Stranded Total RNA with Ribo-Zero Gold kit (Illumina), designed to remove ribosomal RNA and enhance the representation of RNA species in the sequencing library. Sequencing was performed on an Illumina platform, providing comprehensive coverage of the transcriptome. Shanghai OE Biotech Co., Ltd. (Shanghai, China) conducted the transcriptome sequencing and subsequent bioinformatic analysis. Differentially expressed genes were identified based on a fold change threshold of ≥1.2 and a statistical significance level of P<0.05. For functional interpretation of these differentially expressed genes, Metascape was employed to analyze and visualize their biological functions and associated pathways.

**Cut&Tag analysis**

Cells were collected, quantified, and centrifuged at 1800 g for 5 minutes to pellet. After discarding the supernatant, cells underwent two PBS washes, followed by resuspension in pre-chilled cell freezing medium and gradual freezing. The cells were then attached to magnetic beads coated with streptavidin and permeabilized with digitonin. This preparation facilitated subsequent incubation steps with Concanavalin A (ConA) beads, a primary anti-QRICH1 antibody (A304-934A, Bethyl Laboratories, 1:100 dilution), and a secondary goat anti-rabbit IgG H&L antibody (AB6702, Abcam, 1:1000 dilution), along with high-activity PG-Tn5/PA-Tn5 transposase for tagmentation. Following cell lysis, DNA was extracted, and library preparation was conducted through PCR amplification. Prepared libraries were pooled to achieve the desired concentration for sequencing depth and sequenced using the Illumina PE150 platform. For data analysis, Clean Data were aligned to the reference genome using Bowtie2, with subsequent filtering to remove low-quality alignments, PCR duplicates, and mitochondrial reads. Peaks were identified with MACS3, applying statistical criteria. Data visualization, including scatter plots, correlation plots, and heatmaps, was performed using DeepTools v2.27.1. Peak annotation utilized ChIPseeker v1.12.1 in R, and binding site motifs were identified using MEME-CHIP v5.0.5. Peaks exhibiting an |log2FoldChange|>0.58 was classified as specific.

**Chip qPCR**

Chromatin immunoprecipitation (ChIP) was conducted on NRCMs using a commercial kit (Thermo Fisher Scientific), according to the manufacturer's protocol. Initially, chromatin was cross-linked using 1% formaldehyde (Thermo Fisher Scientific) and subsequently quenched with 0.125 M glycine for 5 minutes. Following cross-linking, cells underwent lysis, sonication to shear DNA to an optimal size, and immunoprecipitation. The QRICH1 antibody (A304-934A, Bethyl Laboratories, used at a 1:100 dilution) and a control IgG antibody were employed for immunoprecipitation. The DNA associated with the precipitated chromatin was purified. The purified DNA was then analyzed by quantitative PCR (qRT-PCR). The sequences of primers are detailed in **Table S6**.

**Luciferase reporter assay**

shQRICH1 H9C2 stable cells or shQRICH1-NC cells were transfected with pcDNA3.1+ATF6-Luc+pRL-TK or QRICH1+ATF6-Luc+pRL-TK using Lipofectamine 3000 (L3000075, Invitrogen). Post-transfection, groups were treated with PBS, Tm, or ISO. After 48 hours, cells were lysed, and dual-luciferase activities were quantified using the Dual-Luciferase® Reporter Assay System (Promega) on a SpectraMax i3 luminometer (Molecular Devices).

**qRT-PCR**

RNA was isolated from collected cells using VeZol Reagent (Vazyme Biotech), as per the manufacturer's protocol. cDNA was synthesized from the RNA with the PrimeScript™ RT Reagent Kit with gDNA Eraser (Takara), followed by a 10-fold dilution in deionized water. The diluted cDNA, specific primers, and ChamQ Universal SYBR qRT-PCR Master Mix (Vazyme Biotech) were combined to prepare PCR samples. Quantitative PCR analysis was conducted using the FTC-3000P system (Funglyn Biotech).

**Western-Blot**

Total protein was extracted from cardiac tissue using a tissue homogenizer (ZHFB-CL-48) or from cultured cells. Proteins were then separated by SDS-PAGE and transferred to PVDF membranes (Millipore). These membranes were incubated overnight at 4°C with primary antibodies specific to the proteins of interest, followed by washing with PBST. Incubation with HRP-conjugated secondary antibodies was done at room temperature for 1 hour. Protein bands were detected using an automated chemiluminescence imaging system (Clinx Science).

**References**

1. Schmidt EK, Clavarino G, Ceppi M, Pierre P (2009) SUnSET, a nonradioactive method to monitor protein synthesis. Nature Methods 6:275-277 doi:10.1038/nmeth.1314

2. Ravi V, Jain A, Mishra S, Sundaresan NR (2020) Measuring Protein Synthesis in Cultured Cells and Mouse Tissues Using the Non‐radioactive SUnSET Assay. Current Protocols in Molecular Biology 133 doi:10.1002/cpmb.127

**Supplemental Figures and Figure Legends**

**
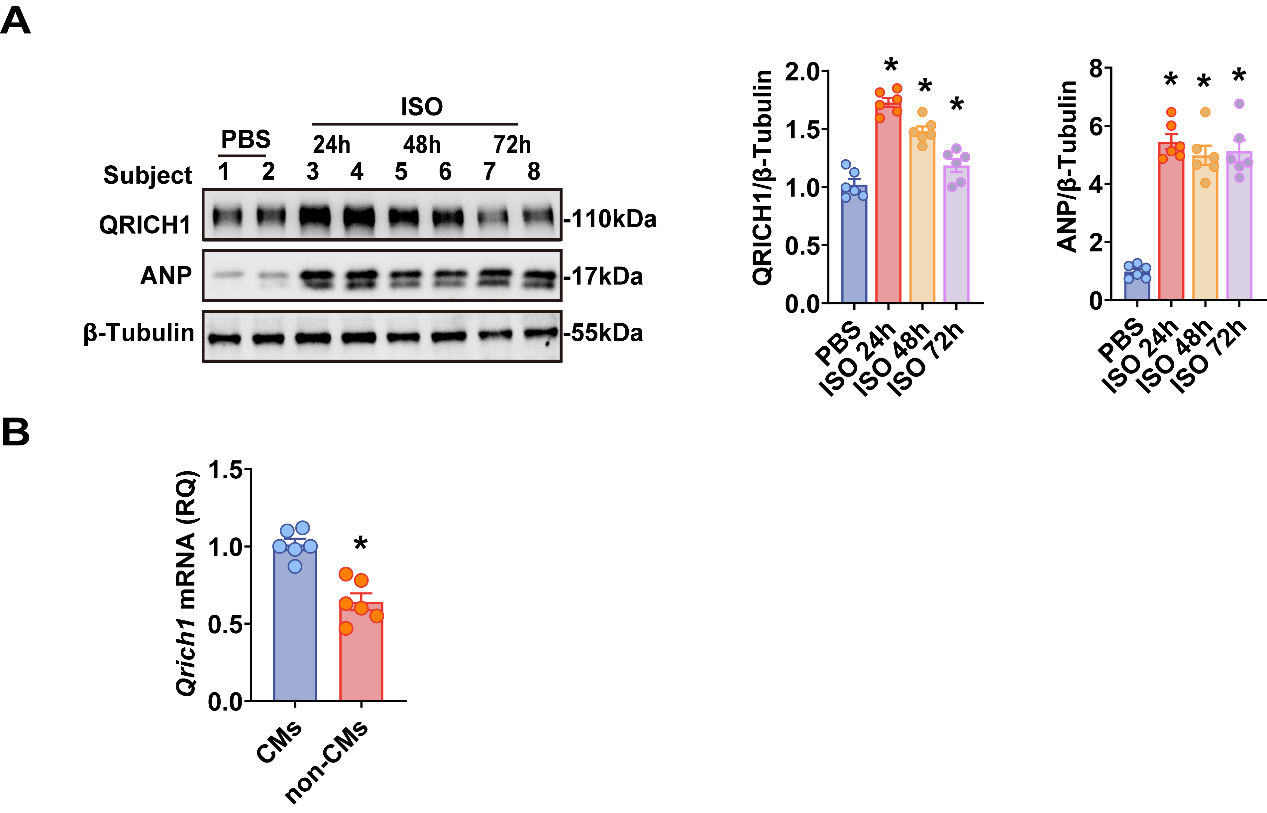
**

**Supplementary Fig. 1** **QRICH1 expression is upregulated in hypertrophic cardiomyocytes. A**, Western blots (**left**) and quantification (**right**) of QRICH1, ANP levels in neonatal rat cardiomyocytes (NRCMs) treated with phosphate buffer saline (PBS) or isoproterenol (ISO; 1 μmol/L) for 24, 48, and 72 h (n=6 independent experiments). **B**, qRT-PCR analysis of *Qrich1* mRNA expression in the cardiomyocytes and non-cardiomyocytes (n=6 independent experiments). **P*<0.05 compared with respective controls. Data are expressed as means ± SEM; unpaired two-tailed Student’s *t*-test or one-way ANOVA with post-hoc multiple comparisons.

**
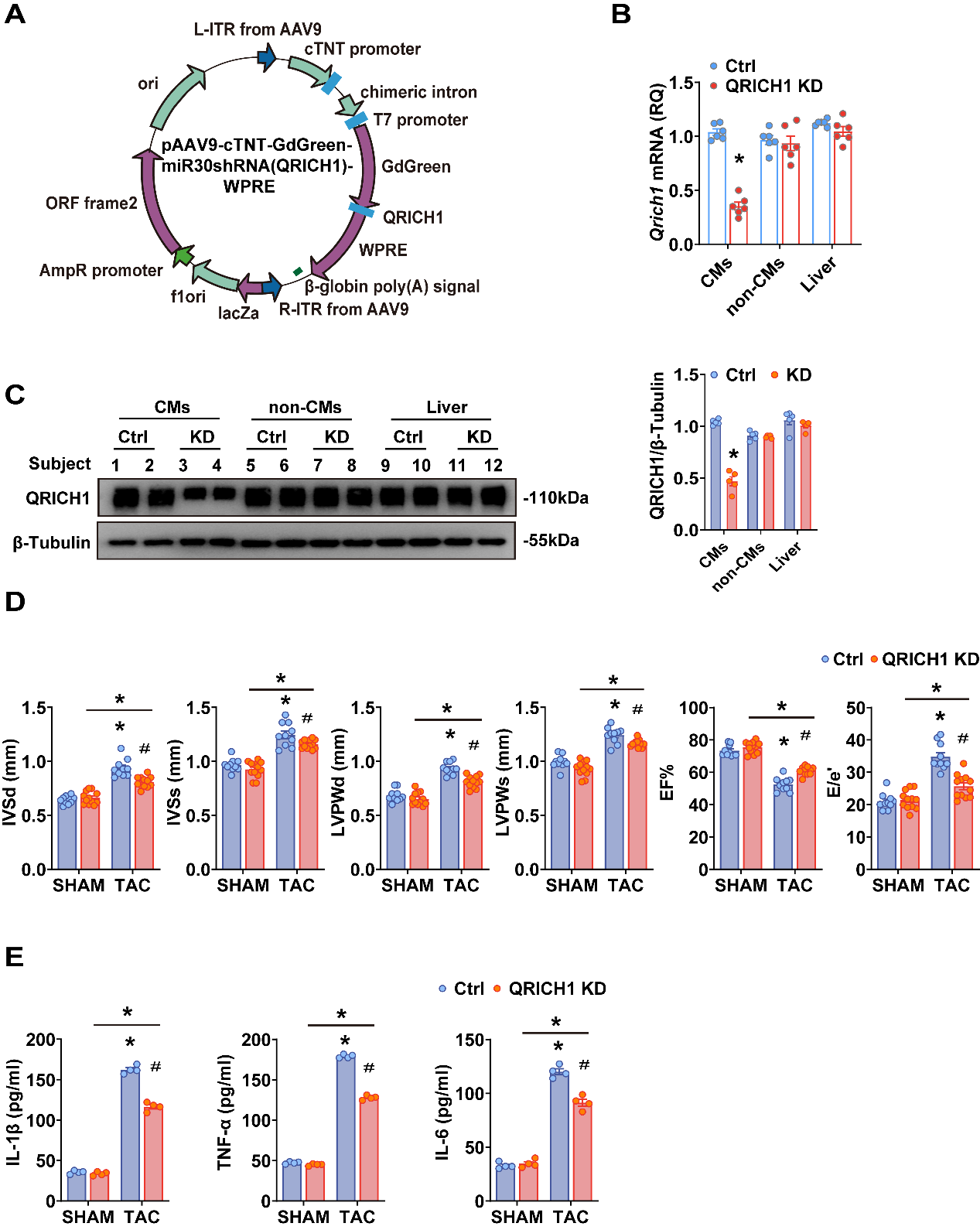
**

**Supplementary Fig. 2 Cardiac-specific knockdown expression of QRICH1 prevents cardiac dysfunction induced by TAC. A**, Construction of QRICH1 KD AAV9 vector targeting mice heart. **B and C**, The knockdown efficiency of QRICH1 mRNA (B) or protein expression (C) in mouse cardiomyocytes, non-cardiomyocytes and liver (n=5 mice per group; **P*<0.05 compared to CMs/Ctrl group). **D**, Echocardiographic measurements of Interventricular end-diastolic septum thickness (IVSd), Interventricular end-systolic septum thickness (IVSs), LV end-diastolic posterior wall thickness (LVPWd), LV end-systolic posterior wall thickness (LVPWs), ejection fraction (EF) and the ratio of peak early transmitral flow velocity to the peak early diastolic mitral annular velocity (E/e’) in Ctrl and QRICH1 KD mice 4 weeks after sham or TAC surgery (n=10-12 mice per group). **E**, Measurement levels of IL-1β, TNF-α and IL-6 determined by ELISA (n=4 mice per group). **P*<0.05 compared to Ctrl/SHAM group, or the value shown by the bar. ^#^*P*<0.05 compared to Ctrl/TAC group. Data are presented as mean ± SEM. **B and C**, unpaired two-tailed Student’s *t*-test. **D** and **E**, 2-way ANOVA followed by Bonferroni post-test or Tukey post-test.

**
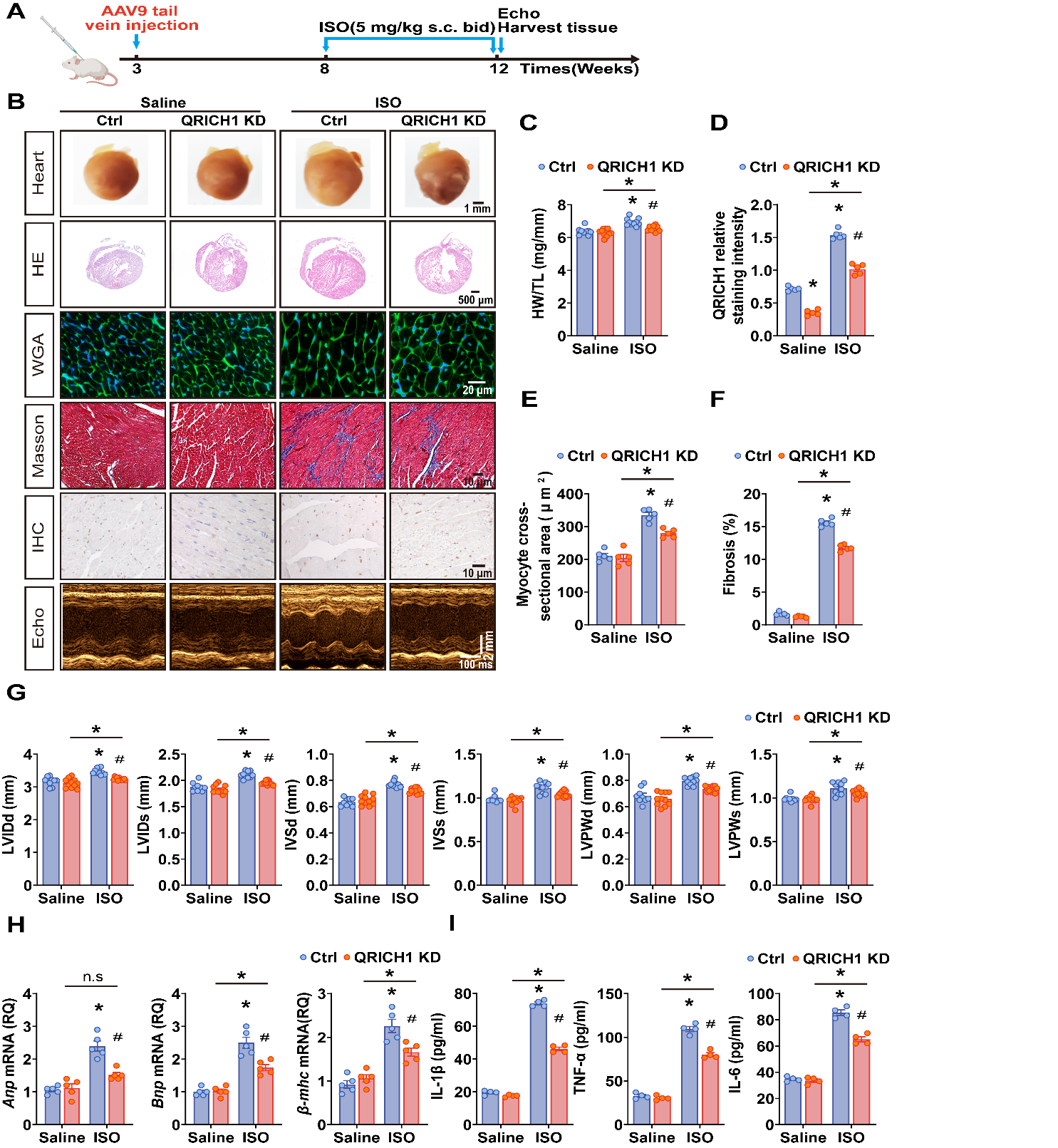
**

**Supplementary Fig. 3 Knocking down QRICH1 alleviates myocardial hypertrophy and preserves cardiac function induced by ISO.** **A**, Schematic timeline of ISO-induced cardiac remodeling in QRICH1 KD mice compared to Ctrl. Adeno-associated virus of serotype 9 (AAV9) containing either GFP or shQRICH1 targeting cardiomyocytes was injected into mice at a concentration of 5×10^11^ (genome copies/mL) for 5 weeks. Five weeks later, the ISO procedure was performed. **B**, Representative gross morphology of mouse hearts (**the top row**, scale bars=1 mm), cross-sections of the heart stained with Hematoxylin and Eosin (**the second row**, scale bars=500 μm), cell boundaries stained with wheat germ agglutinin (**the third row**, scale bars=20 μm), LV fibrosis stained with Masson's trichrome (**the forth row**, scale bars=10 μm), LV QRICH1 expression determined by immunohistochemistry (**the fifth row**, scale bars=10 μm), M-mode echocardiography images of the LV chamber in QRICH1 KD and Ctrl littermate mice 4 weeks after Saline or ISO treatment (**the bottom row**, scale bars=2 mm). **C**, The ratio of heart weight to tibia length (HW/TL) (n=9,11,10,12 mice from left to right). **D**, Statistical results for QRICH1 immunohistochemistry expression (n=5 mice per group). **E**, Quantification of cell cross-sectional area (n=5 mice per group). **F**, Quantification of myocardial interstitial collagen (n=5 mice per group). **G**, Echocardiographic measurements of LV end-diastolic internal diameter (LVIDd), LV end-systolic internal diameter (LVIDs), Interventricular end-diastolic septum thickness (IVSd), Interventricular end-systolic septum thickness (IVSs), LV end-diastolic posterior wall thickness (LVPWd) and LV end-systolic posterior wall thickness (LVPWs) in Ctrl and QRICH1 KD mice 4 weeks after Saline or ISO treatment (n=9-12 mice per group). **H**, Measurement levels of myocardial hypertrophy-associated transcripts ANP (atrial natriuretic peptide), BNP (brain natriuretic peptide), β-MHC (β myosin heavy chain) (n=5 mice per group). **I**, Measurement levels of IL-1β, TNF-α and IL-6 determined by ELISA (n=4 mice per group). **P*<0.05 compared to Ctrl/Saline group or the value shown by the bar. ^#^*P*<0.05 compared to Ctrl/ISO group. n.s. indicates no signifcant difference. Data are presented as mean ± SEM. 2-way ANOVA followed by Bonferroni post-test or Tukey post-test.

**
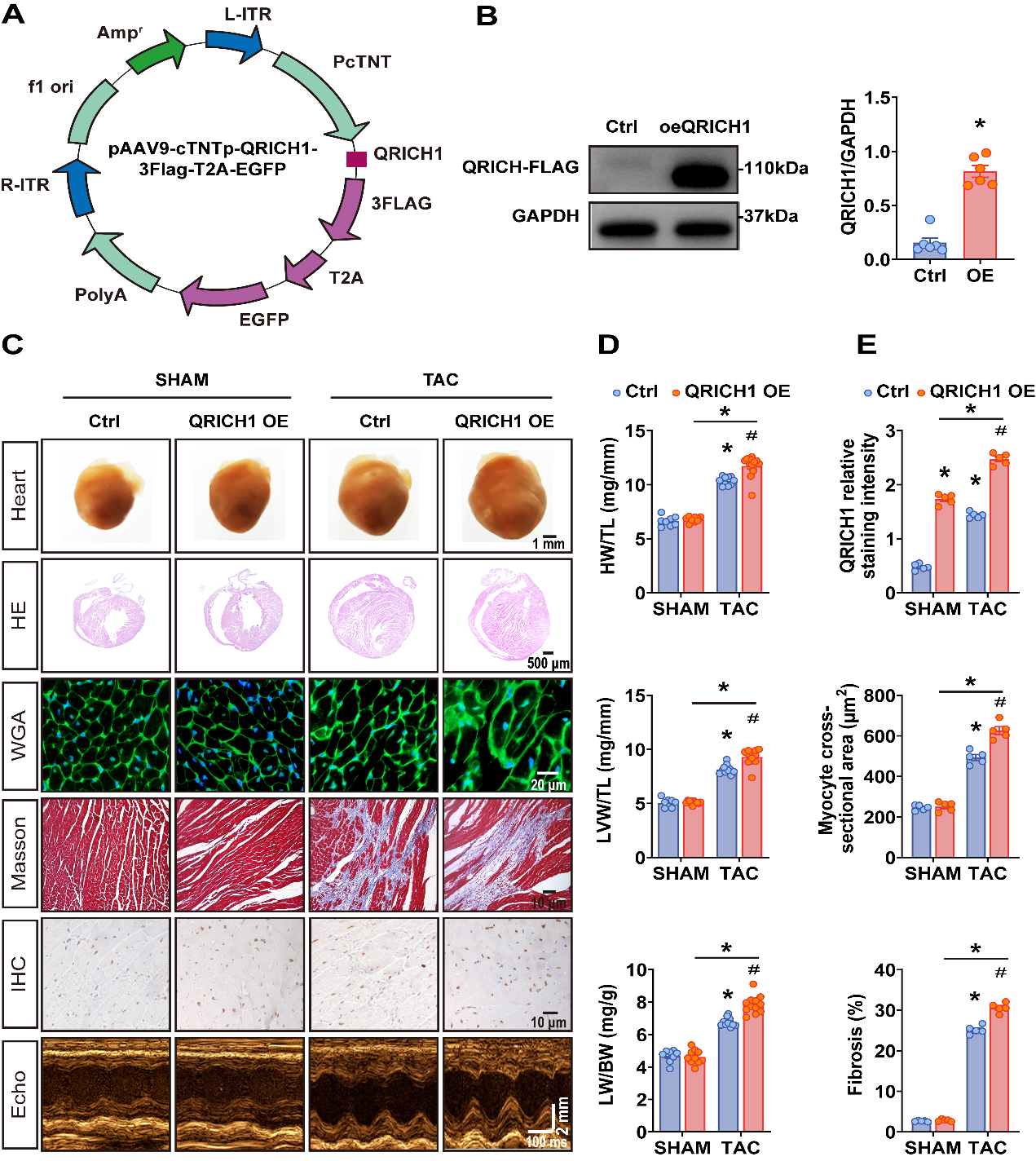
**
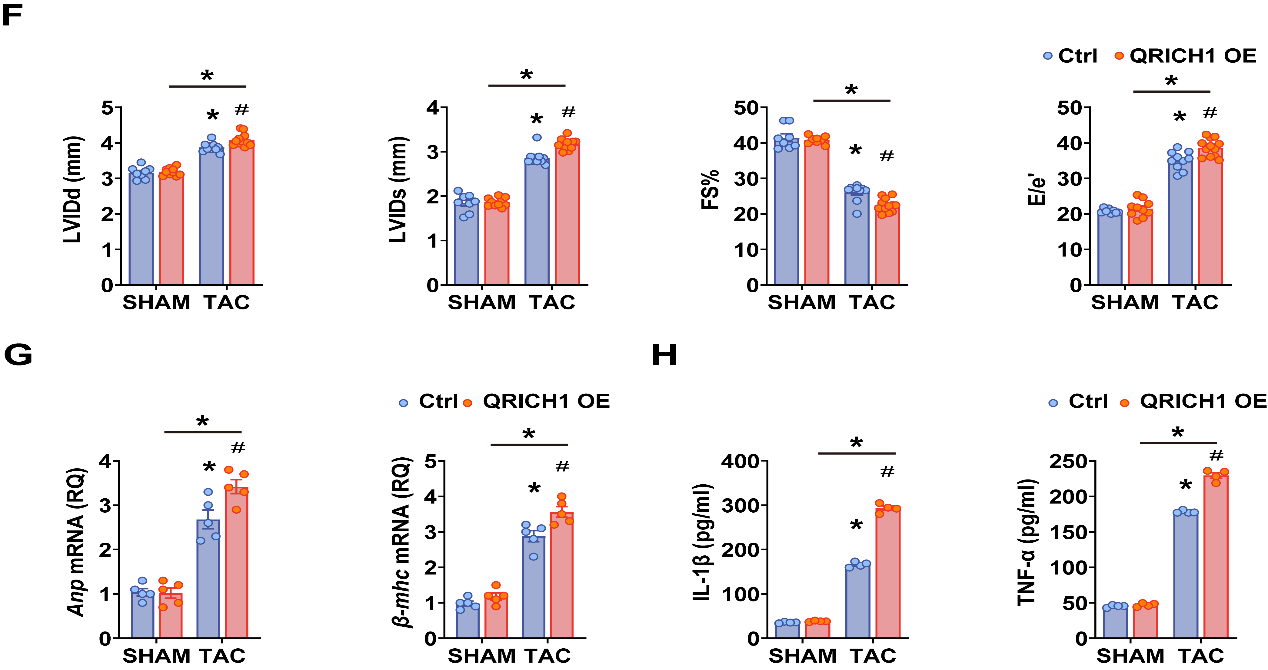
**
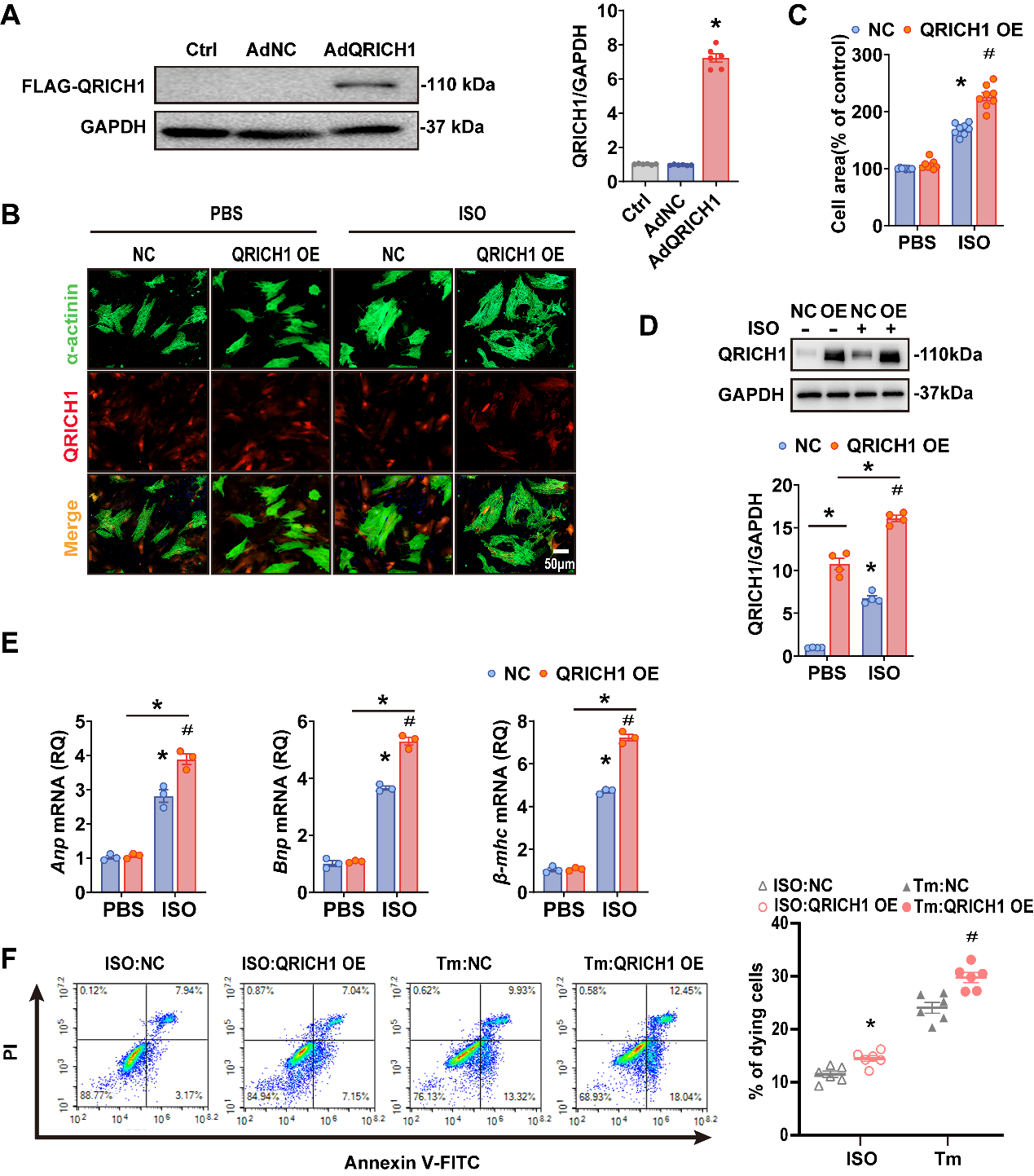
**

**Supplementary Fig. 4** **QRICH1 overexpression exacerbates TAC-induced cardiac hypertrophy and heart failure. A**, Construction of QRICH1 OE AAV9 vector targeting mice heart. **B**, The overexpression efficiency of QRICH1 in mouse hearts. (n=6 mice per group; **P*<0.05 compared to Ctrl group). **C**, Representative gross morphology of mouse hearts (**the top row**, scale bars=1 mm), cross-sections of the heart stained with Hematoxylin and Eosin (**the second row**, scale bars=500 μm), cell boundaries stained with wheat germ agglutinin (**the third row**, scale bars=20 μm), LV fibrosis stained with Masson's trichrome (**the forth row**, scale bars=10 μm), LV QRICH1 expression determined by immunohistochemistry (**the fifth row**, scale bars=10 μm), M-mode echocardiography images of the LV chamber in QRICH1 OE and Ctrl littermate mice subjected to sham or TAC surgery (**the bottom row**, scale bars=2 mm) **D**, The ratio of heart weight to tibia length (HW/TL), left ventricle weight to tibia length (LVW/TL) and lung weight to body weight (LW/BW) (n=8,10,10,12 mice from left to right). **E**, Statistical results for QRICH1 immunohistochemistry expression, quantification of cell cross-sectional area and myocardial interstitial collagen (n=5 mice per group). **F**, Echocardiographic measurements of LV end-diastolic internal diameter (LVIDd), LV end-systolic internal diameter (LVIDs), fractional shortening (FS) and the ratio of peak early transmitral flow velocity to the peak early diastolic mitral annular velocity (E/e’) in Ctrl and QRICH1 OE mice 4 weeks after sham or TAC surgery (n=8-12 mice per group). **G**, Measurement levels of myocardial hypertrophy-associated transcripts ANP (atrial natriuretic peptide) and β-MHC (β myosin heavy chain) (n=5 mice per group). **H**, Measurement levels of IL-1β and TNF-α determined by ELISA (n=4 mice per group). **P*<0.05 compared to Ctrl/SHAM group or the value shown by the bar. ^#^*P*<0.05 compared to Ctrl/TAC group. Data are presented as mean ± SEM. **B**, unpaired two-tailed Student’s *t*-test. **D**-**H**, 2-way ANOVA followed by Bonferroni post-test or Tukey post-test.


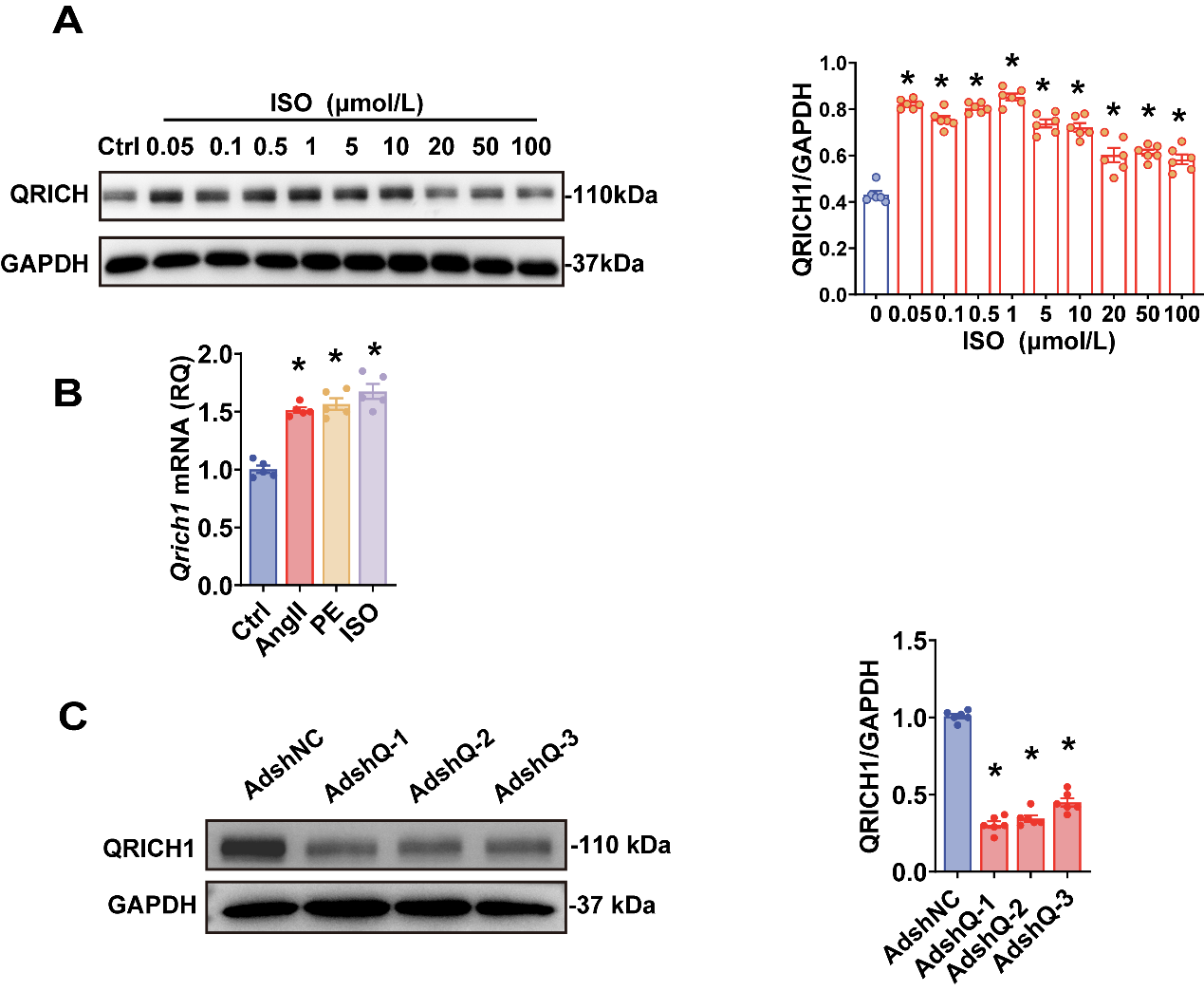


**Supplementary Fig. 5 QRICH1 expression is upregulated under various hypertrophic stimuli. A**, Western blots (**left**) and quantification (**right**) of QRICH1 expression in neonatal rat primary cardiomyocytes (NRCMs) treated with different doses of ISO for 24h (n=6 independent experiments). **B**, Quantitative qRT-PCR analysis of QRICH1 mRNA expression in different hypertrophic stimuli (n=5 samples per group). **C**, Western blots (**left**) and quantification (**right**) of QRICH1 knockdown in NRCMs infected with AdshNC or AdshQRICH1-1/2/3 (n=4 samples per group). **P*<0.05 compared to control (Ctrl/NC) group. Data are presented as mean ± SEM, unpaired two-tailed Student’s *t*-test.

**
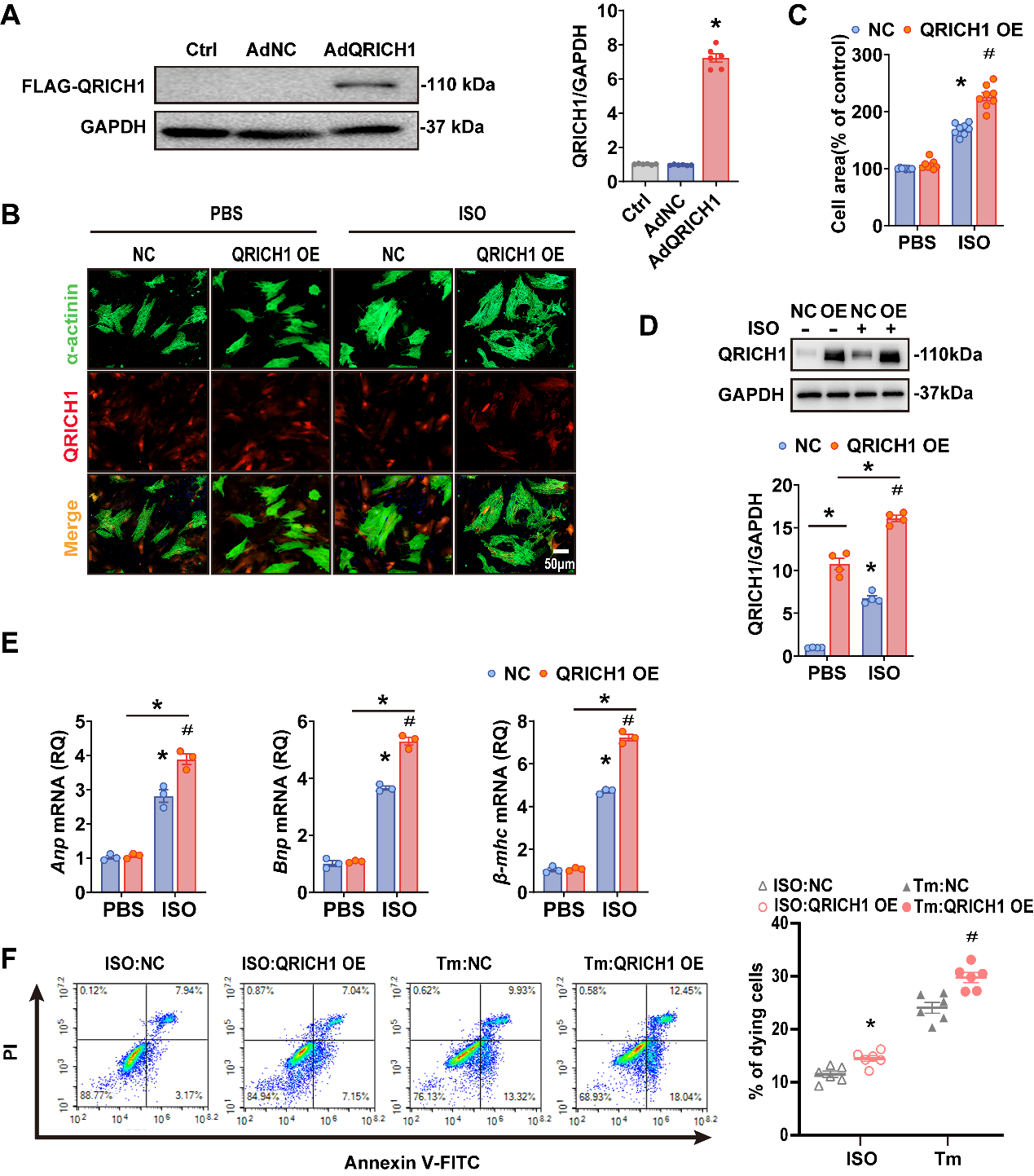
**

**Supplementary Fig. 6 QRICH1 exacerbates ISO-induced cardiomyocyte hypertrophy in vitro**. **A**, Western blots (**left**) and quantification (**right**) of overexpression QRICH1 in Neonatal rat cardiomyocytes (NRCMs) infected with AdNC or AdQRICH1 (n=6 independent experiments, **P*<0.05 compared to Ctrl group). **B**, Representative immunofluorescence images of α-actinin (green), QRICH1 (red) and DAPI (4’,6-diamidino-2-phenylindole; blue) staining in NRCMs infected with adenoviruses expressing AdGFP or AdQRICH1 and treated with phosphate-buffered saline (PBS) or isoproterenol for 48h. Scale bars=50 μm. **C**, Quantification of relative cell surface areas of NRCMs infected with indicated adenoviruses in response to PBS or ISO treatment (n>100 cells per group; **P*<0.05 compared to PBS/NC group; ^#^*P*<0.05 compared to ISO/NC group). **D**, Western blots (**upper**) and quantification (**lower**) of QRICH1 expression in NRCMs infected with AdGFP or AdQRICH1 (n=4 independent experiments; **P*<0.05 compared to PBS/NC group or the value shown by the bar; ^#^*P*<0.05 compared to ISO/NC group). **E**, Relative mRNA levels of ANP (atrial natriuretic peptide), BNP (brain natriuretic peptide), β-MHC (β myosin heavy chain) in AdGFP- or AdQRICH1- infected NRCMs 48h after PBS or ISO treatment (n=3 independent experiments; **P*<0.05 compared to PBS/NC group or the value shown by the bar; ^#^*P*<0.05 compared to ISO/NC group). **F**, Representative fluorescence-activated cell sorting (FACS) analysis (left) and Measurement of dying (PI or Annexin V positive) cells treated with isoproterenol (ISO) or tunicamycin (Tm) for 72 h (n=6 independent experiments; **P*<0.05 compared to ISO/NC group; ^#^*P*<0.05 compared to TM/NC group). Data are presented as mean ± SEM. **C**, **D** and **E**, 2-way ANOVA followed by Bonferroni post-test or Tukey post-test. **A** and **F**, unpaired two-tailed Student’s *t*-test.


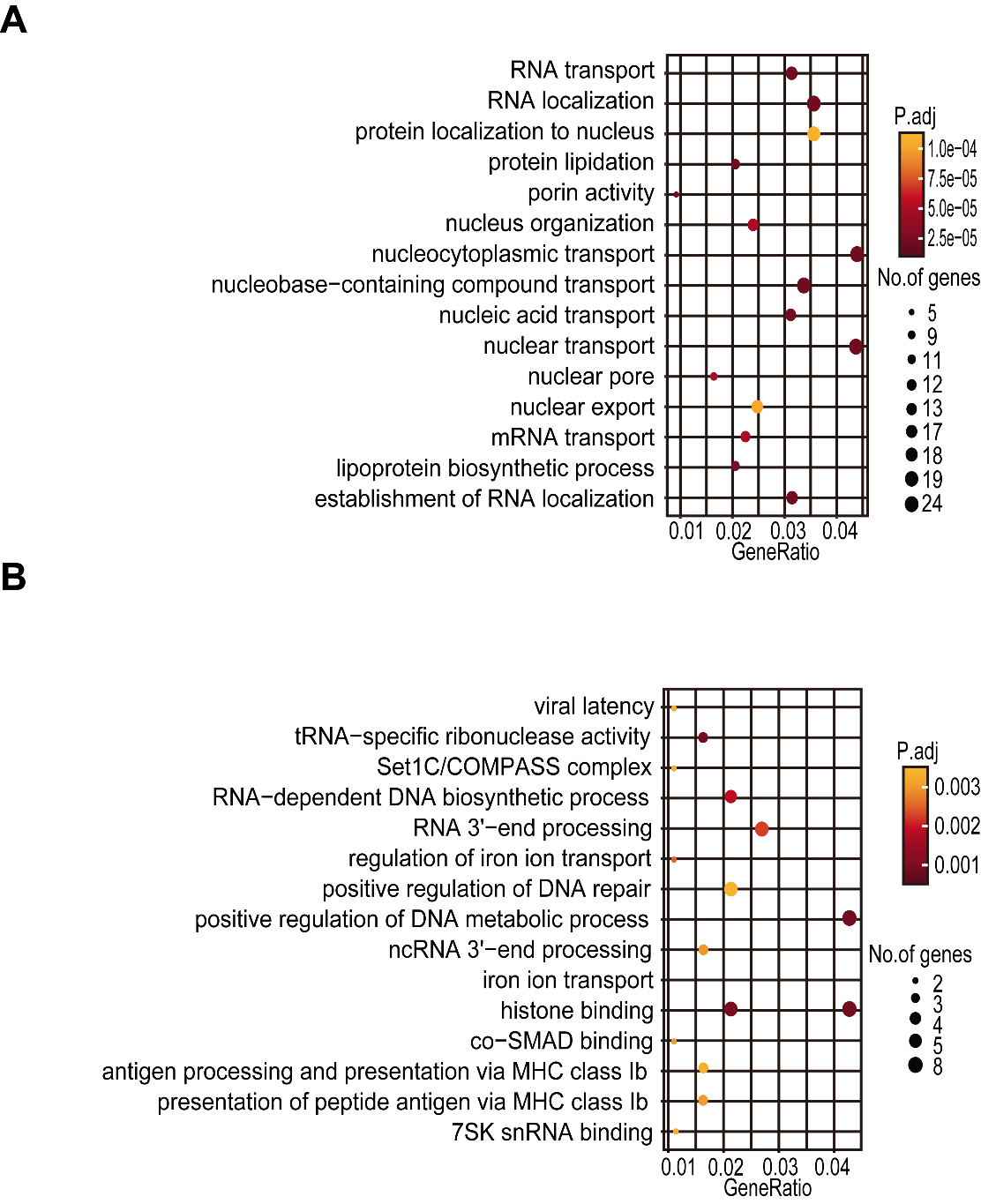


**Supplementary Fig. 7 Gene ontology (GO) enrichment analysis of differentially peaked (DP) promoter genes in Ctrl versus QRICH1 KD cells.** **A**, Gain DP promoter gene GO enrichment. **B**, Loss DP promoter gene GO enrichment.

**
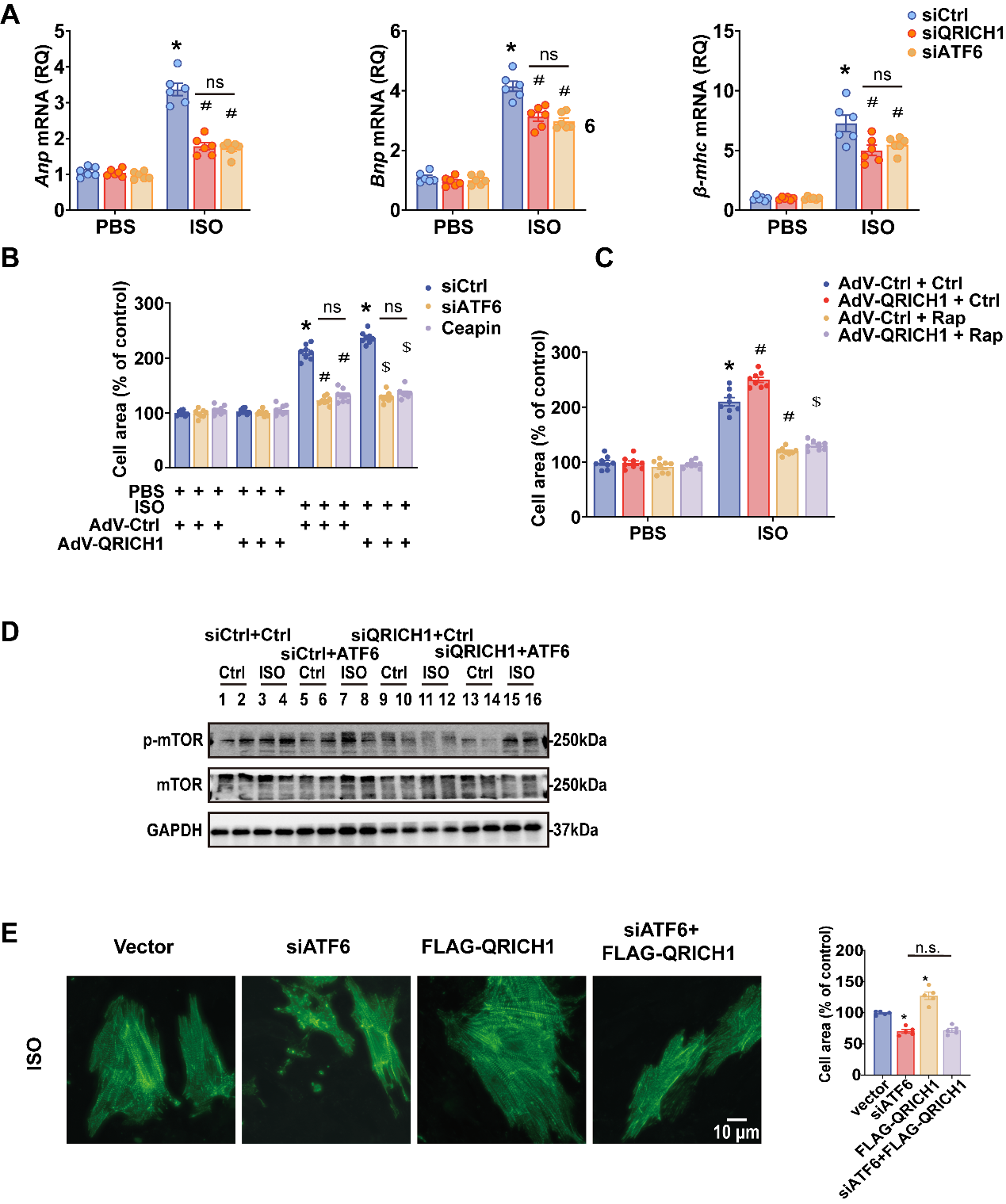
**

**Supplementary Fig. 8 Knockdown of QRICH1 or ATF6 alleviated the ISO-induced cardiomyocyte hypertrophy. A**, Relative mRNA levels of ANP (atrial natriuretic peptide), BNP (brain natriuretic peptide), β-MHC (β myosin heavy chain) in NRCMs infected with siCtrl or siQRICH1 or siATF6 and treated with PBS or isoproterenol (ISO, 1 μM) for 48 h (n=6 independent experiments, **P*<0.05 compared to PBS/siCtrl group; ^#^*P*<0.05 compared to ISO/siCtrl group; n.s. indicates no signifcant difference). **B**, Quantification of relative cell surface areas of NRCMs infected with adenoviruses encoding AdCtrl or AdQRICH1, and either siCtrl or siATF6 or Ceapin (20 μM), followed by treatment ± ISO for 48 h (n>100 cells per group; **P*<0.05 compared to respective siCtrl group; ^#^*P*<0.05 compared to ISO/AdV-Ctrl/siCtrl group; ^$^*P*<0.05 compared to ISO/AdV-QRICH1/siCtrl group; n.s. indicates no signifcant difference). **C**, Quantification of relative cell surface areas of NRCMs infected with adenoviruses
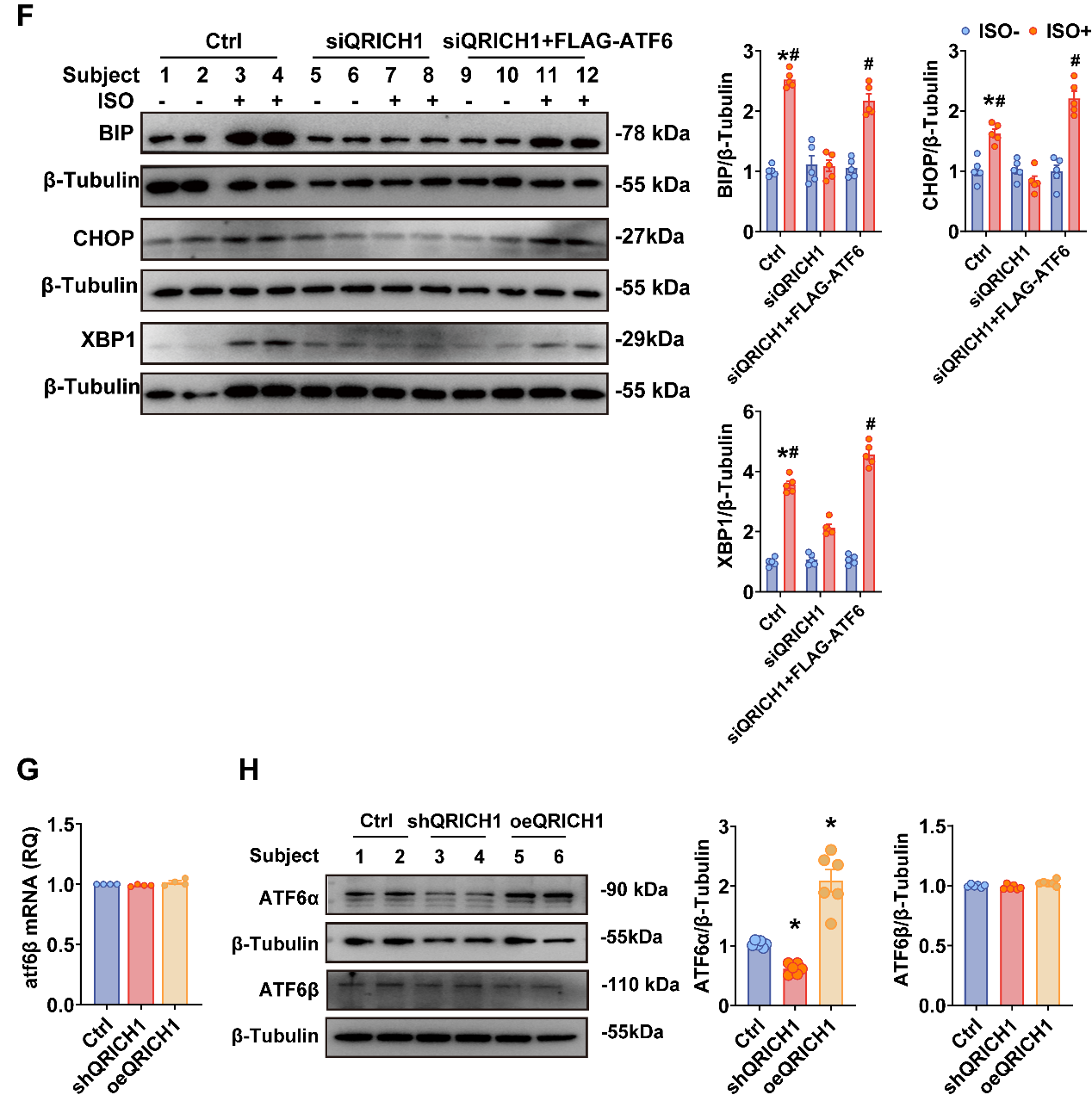
encoding AdCtrl or AdQRICH1, followed by treatment ± ISO with or without rapamycin (Rap, 20 nM) for 48 hours (n>100 cells per group; **P*<0.05 compared to PBS/Adv-Ctrl+Ctrl group; ^#^*P*<0.05 compared to ISO/AdV-Ctrl+Ctrl group; ^$^*P*<0.05 compared to ISO/AdV-QRICH1+Ctrl group). **D**, Western blots of NRCMs infected with a control plasmid or a plasmid encoding Flag-ATF6 and either siCtrl or siQRICH1, followed by treatment ± ISO for 48 h. **E**, Representative immunofluorescence images (**left**) of α-actinin in NRCMs infected with a control plasmid or a plasmid encoding Flag-QRICH1 and either siCtrl or siATF6, followed by treatment ± ISO for 48 h. Measurement (**right**) of cell surface area after ICF, FLAG-positive cells were used for cell surface area analysis (n>100 cells per group, scale bars=10 μm, *P<0.05 compared to vector). **F**， Western blots of canonical ATF6 targets (left) and quantification (right). **G** and **H**, Atf6α/β mRNA (G) and ATF6α/β protein (H) levels in QRICH1-KD/OE NRCMs. *P<0.05 compared to Ctrl. Data are presented as mean ± SEM. 2-way ANOVA followed by Bonferroni post-test or Tukey post-test.


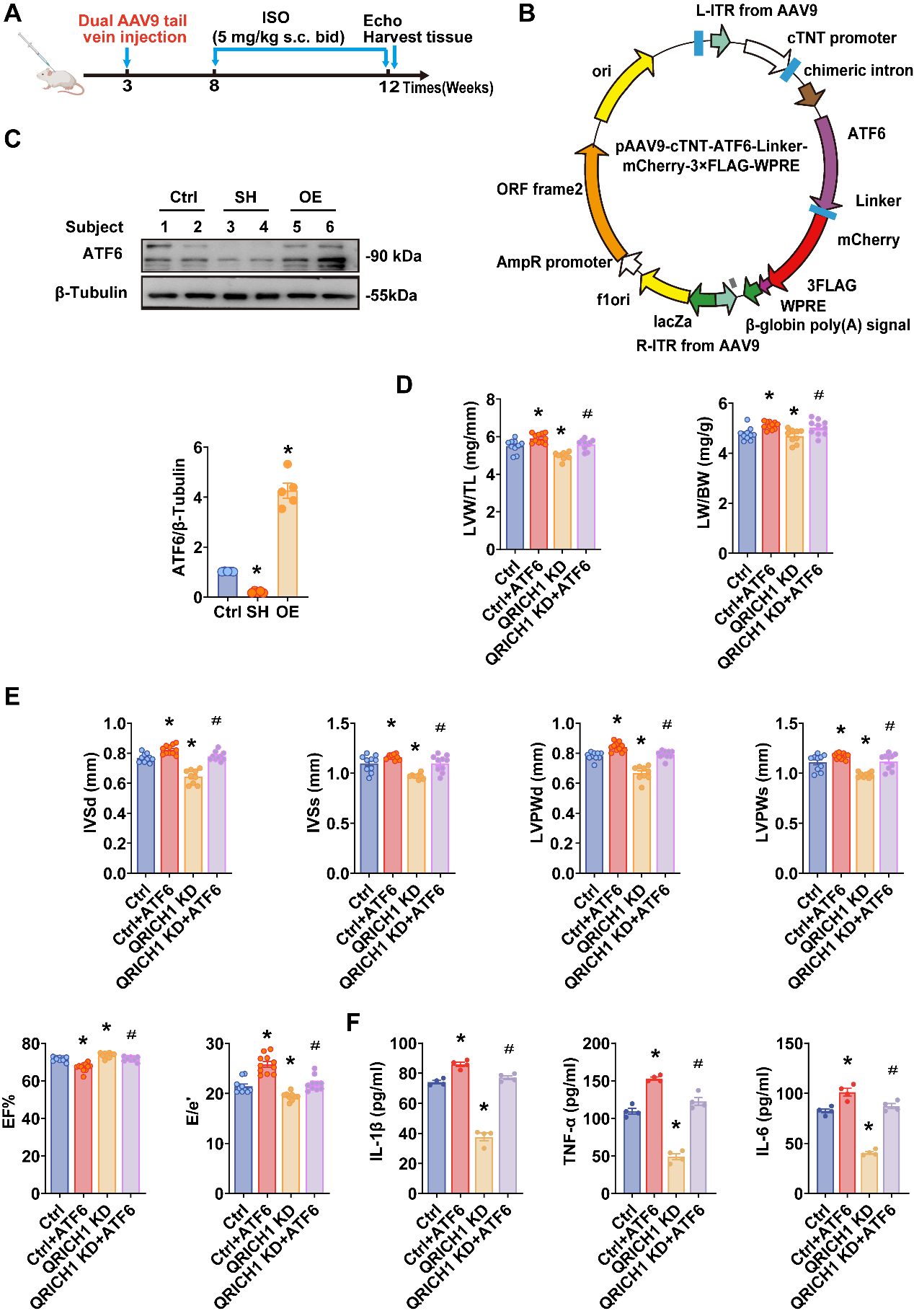


**Supplementary Fig. 9 The impact of cardiomyocyte-specific ectopic ATF6 expression on heart structure and function in QRICH1 knockdown（KD）mice induced by ISO. A**, Schematic timeline of ISO-induced cardiac remodeling in ATF6 OE/QRICH1 KD/ ATF6 OE+QRICH1 KD mice compared to Ctrl. Adeno-associated virus of serotype 9 (AAV9) containing GFP or Flag-ATF6 or shQRICH1 or Flag-
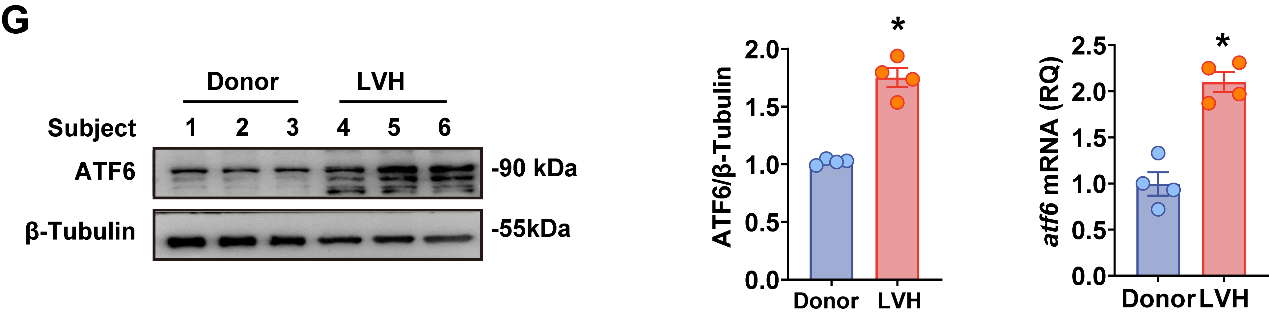
ATF6+shQRICH1 targeting cardiomyocytes was injected into mice at a concentration of 5×10^11^ (genome copies/mL) for 5 weeks. Five weeks later, the ISO procedure was performed. **B**, Construction of an ATF6 overexpression (OE) AAV9 vector targeting the mouse heart. **C**, Western blot analysis (up) and quantification (lower) to confirm ATF6 knockdown （SH） and overexpression （OE）using our AAV approach. **D**, The ratio of left ventricle weight to tibia length (LVW/TL) and lung weight to body weight (LW/BW) (n=10, 11, 9, 10 mice from left to right). **E**, Echocardiographic measurements of Interventricular end-diastolic septum thickness (IVSd), Interventricular end-systolic septum thickness (IVSs), LV end-diastolic posterior wall thickness (LVPWd), LV end-systolic posterior wall thickness (LVPWs), ejection fraction (EF) and the ratio of peak early transmitral flow velocity to the peak early diastolic mitral annular velocity (E/e’) in Ctrl/ATF6 OE/QRICH1 KD/ ATF6 OE+QRICH1 KD mice 4 weeks after ISO treatment (n=9-11 mice per group). **F**, Measurement levels of IL-1β, TNF-α and IL-6 determined by ELISA (n=4 mice per group). **G**, Western blots (left) and quantification (right) of ATF6 levels in normal donor hearts (n=4) and hearts collected from LVH patients (n=4). **P*<0.05 compared to Ctrl or Donor group. ^#^*P*<0.05 compared to QRICH1 KD group. Data are presented as mean ± SEM. unpaired two-tailed Student’s *t*-test.

**
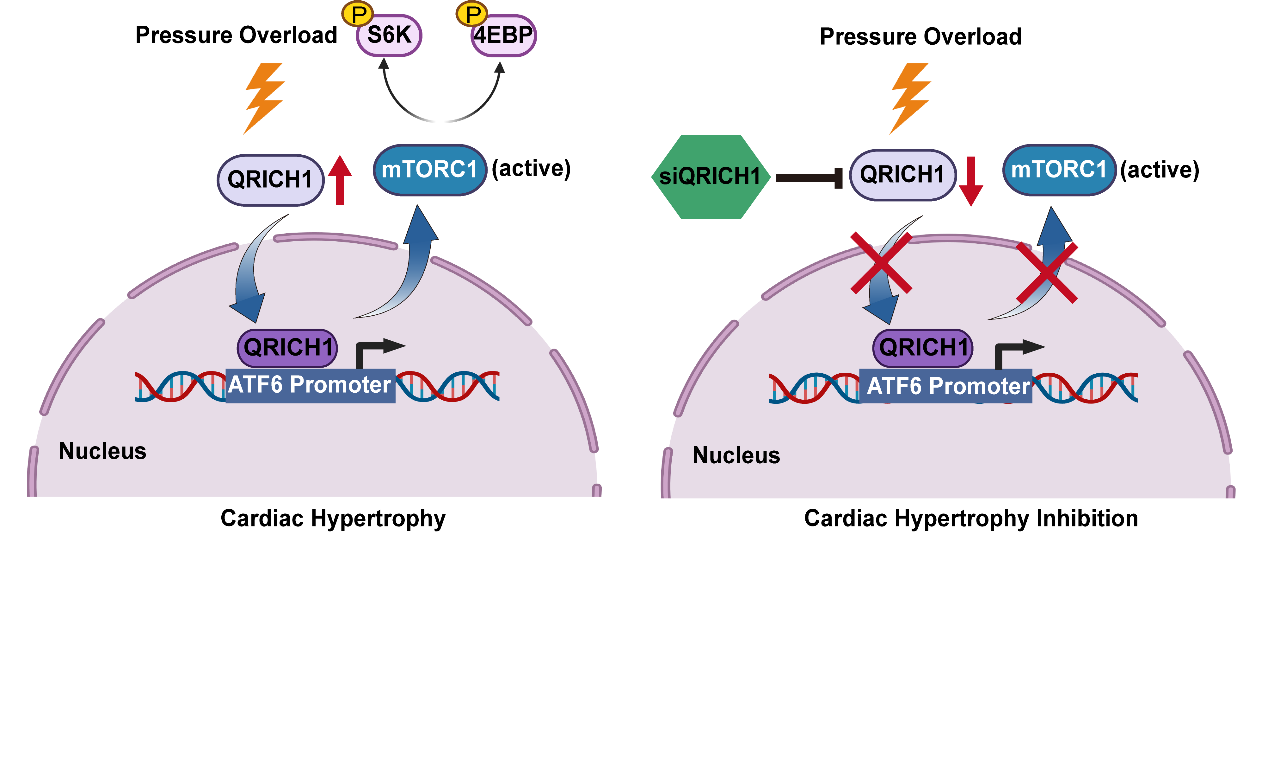
**

**Supplementary Fig. 10** **QRICH1 promotes cardiac hypertrophy by interacting with ATF6 (left), and inhibition of QRICH1 attenuates cardiac remodeling (right).**

**Supplemental Tables**

**Supplementary Table 1.** Human subject characteristics

| **Subject** | **Diagnosis** | **Age** | **Gender** | **LVPWd(mm)** | **IVSd(mm)** |
| --- | --- | --- | --- | --- | --- |
| 1 | donor | 37 | male | N/A | N/A |
| 2 | donor | 52 | male | N/A | N/A |
| 3 | donor | 49 | female | N/A | N/A |
| 4 | donor | 52 | female | N/A | N/A |
| 5 | LVH, HTN | 38 | male | 15 | 15 |
| 6 | LVH, HTN, CAD | 47 | male | 8.1 | 14.2 |
| 7 | LVH, MR, CAD | 56 | female | 9.5 | 18.1 |
| 8 | LVH, HTN | 67 | female | 12.3 | 15.9 |

LVH: Left ventricular hypertrophy; HTN: Hypertension; CAD: coronary artery disease; MR: Mitral Regurgitation; LVPWd: LV end-diastolic posterior wall thickness; IVSd: Interventricular end-diastolic septum thickness; N/A: not available

**Supplementary Table 2.** Echocardiography and anatomical evaluations of QRICH1 KD mice and non-transgenic controls (Ctrl) following 4 weeks of sham or TAC surgery

| Treatment | SHAM | | TAC | |
| --- | --- | --- | --- | --- |
| Groups | Ctrl | QRICH1 KD | Ctrl | QRICH1 KD |
| Number | n=10 | n=12 | n=10 | n=12 |
| BW (g) | 27.12±0.22 | 26.73±0.17 | 26.8±0.34 | 27.08±0.21 |
| HW (mg) | 124.9±2.25 | 119±2.15 | 195.3±5.47* | 166.67±4.49*† |
| HW/BW(mg/g) | 4.6±0.08 | 4.46±0.09 | 7.29±0.16* | 6.16±0.17*† |
| LW (mg) | 128.4±2.54 | 124.25±2.73 | 185.9±2.62* | 163.3±3.21 |
| LW/BW(mg/g) | 4.74±0.1 | 4.66±0.12 | 6.95±0.11* | 6.04±0.13*† |
| TL (mm) | 18.86±0.11 | 18.92±0.27 | 19.1±0.17 | 19.12±0.22 |
| HW/TL(mg/mm) | 6.62±0.13 | 6.3±0.12 | 10.23± 0.3* | 8.74±0.28*† |
| LVW(mg) | 96.8±2.77 | 94.08±2.71 | 149.7±5.51 | 128.58±4.82 |
| LVW/TL(mg/mm) | 5.13±0.15 | 4.98±0.14 | 7.85±0.31 | 6.74±0.28 |
| HR(bmp) | 489±5 | 472±6 | 468±14* | 488±9 |
| IVSd(mm) | 0.64±0.01 | 0.66±0.02 | 0.94±0.03* | 0.81±0.02*† |
| IVSs(mm) | 0.97±0.02 | 0.93±0.02 | 1.25±0.04* | 1.16±0.01*† |
| LVIDd(mm) | 3.17±0.03 | 3.13±0.04 | 3.89±0.04* | 3.51±0.03*† |
| LVIDs(mm) | 1.82±0.05 | 1.74±0.03 | 2.9±0.06* | 2.4±0.04*† |
| LVPWd(mm) | 0.68±0.02 | 0.65±0.02 | 0.94±0.02* | 0.81±0.02*† |
| LVPWs(mm) | 1.0±0.02 | 0.93±0.02 | 1.24±0.02* | 1.17±0.01*† |
| FS(%) | 41.17±0.94 | 42.98±0.87 | 25.55±0.9* | 31.51±0.54*† |
| EF(%) | 73.35±1.09 | 75.4±1.0 | 52.49±1.33* | 60.98±0.73*† |
| E/e’ | 20.95±0.8 | 21.47±0.82 | 34.79±1.31* | 25.67±1.03*† |

Body weight(BW), Heart weight(HW), Left ventricular weight(LVW), Lung weight(LW), Tibial length(TL), Heart rates(HR), Interventricular end-diastolic septum thickness(IVSd), Interventricular end-systolic septum thickness (IVSs), Left ventricular end-diastolic internal diameter (LVIDd), LV end-systolic internal diameter (LVIDs), LV end-diastolic posterior wall thickness(LVPWd), LV end-systolic posterior wall thickness (LVPWs), Fractional shortening (FS), Ejection fraction(EF), The ratio of peak early transmitral flow velocity to the peak early diastolic mitral annular velocity(E/e’), transverse aortic constriction (TAC). Data are presented as mean ± SEM. Statistical significance was determined by Two-way ANOVA with interaction analysis and Tukey or Bonferroni post-test. **P*<0.05 compared to SHAM/Ctrl group; ^†^*P* <0.05 compared to TAC/Ctrl group.

**Supplementary Table 3.** Echocardiography and anatomical evaluations of QRICH1 KD mice and non-transgenic controls (Ctrl) following 4 weeks of saline or ISO injection

| Treatment | Saline | | ISO | |
| --- | --- | --- | --- | --- |
| Groups | Ctrl | QRICH1 KD | Ctrl | QRICH1 KD |
| Number | n=9 | n=11 | n=10 | n=12 |
| BW (g) | 27.37±0.25 | 27.19±0.24 | 27.18±0.37 | 27.08±0.22 |
| HW (mg) | 121.00±1.38 | 119.09±1.19 | 132.8±1.54* | 127.75±1.19*† |
| HW/BW(mg/g) | 4.81±0.12 | 4.74±0.08 | 7.82±0.22* | 6.59±0.2*† |
| LW (mg) | 127.67±2.74 | 127.18±2.59 | 130.00±2.21 | 127.92±1.32 |
| LW/BW(mg/g) | 4.67±0.11 | 4.68±0.12 | 4.79±0.08 | 4.73±0.04 |
| TL (mm) | 18.96±0.11 | 18.87±0.21 | 19.13±0.21 | 19.48±0.12 |
| HW/TL(mg/mm) | 6.38±0.08 | 6.31±0.07 | 6.95± 0.07* | 6.56±0.05*† |
| LVW(mg) | 93.33±1.76 | 93.55±1.2 | 106.8±1.2* | 100.08±0.9*† |
| LVW/TL(mg/mm) | 4.92±0.09 | 4.96±0.06 | 5.59±0.07* | 5.14±0.04† |
| HR(bmp) | 485±8 | 469±10 | 482±10* | 476±13 |
| IVSd(mm) | 0.64±0.01 | 0.65±0.01 | 0.77±0.01* | 0.72±0.01*† |
| IVSs(mm) | 0.99±0.01 | 0.97±0.01 | 1.12±0.02* | 1.04±0.01*† |
| LVIDd(mm) | 3.17±0.05 | 3.11±0.04 | 3.47±0.03* | 3.24±0.01*† |
| LVIDs(mm) | 1.88±0.03 | 1.83±0.02 | 2.1±0.02* | 1.95±0.01*† |
| LVPWd(mm) | 0.68±0.02 | 0.66±0.02 | 0.80±0.01* | 0.73±0.01*† |
| LVPWs(mm) | 0.99±0.01 | 0.99±0.01 | 1.11±0.02* | 1.06±0.01*† |
| FS(%) | 40.8±0.75 | 41.06±0.49 | 39.2±0.42 | 39.85±0.28 |
| EF(%) | 72.91±0.88 | 73.24±0.58 | 71.02±0.51 | 71.81±0.34 |
| E/e’ | 20.28±0.41 | 20.35±0.7 | 20.63±0.55 | 19.93±0.44 |

Body weight(BW), Heart weight(HW), Left ventricular weight(LVW), Lung weight(LW), Tibial length(TL), Heart rates(HR), Interventricular end-diastolic septum thickness(IVSd), Interventricular end-systolic septum thickness (IVSs), Left ventricular end-diastolic internal diameter (LVIDd), LV end-systolic internal diameter (LVIDs), LV end-diastolic posterior wall thickness(LVPWd), LV end-systolic posterior wall thickness (LVPWs), Fractional shortening (FS), Ejection fraction(EF), The ratio of peak early transmitral flow velocity to the peak early diastolic mitral annular velocity(E/e’), isoproterenol (ISO). Data are presented as mean ± SEM. Statistical significance was determined by Two-way ANOVA with interaction analysis and Tukey or Bonferroni post-test. **P*<0.05 compared to Saline/Ctrl group; ^†^*P* <0.05 compared to ISO/Ctrl group.

**Supplementary Table 4.** Echocardiography and anatomical evaluations of QRICH1 OE mice and non-transgenic controls (Ctrl) following 4 weeks of sham or TAC surgery

| Treatment | SHAM | | TAC | |
| --- | --- | --- | --- | --- |
| Groups | Ctrl | QRICH1 OE | Ctrl | QRICH1 OE |
| Number | n=8 | n=10 | n=10 | n=12 |
| BW (g) | 27.00±0.24 | 27.2±0.19 | 26.81±0.26 | 27.19±0.25 |
| HW (mg) | 123.88±2.91 | 126.2±2.05 | 190.6±2.71* | 217.8±5.96*† |
| HW/BW(mg/g) | 4.60±0.13 | 4.64±0.07 | 7.12±0.14* | 8.02±0.24*† |
| LW (mg) | 126.13±3.39 | 125.7±4.2 | 181.3±2.1* | 213.17±4.58 |
| LW/BW(mg/g) | 4.68±0.14 | 4.62±0.14 | 6.77±0.09* | 7.84±0.16*† |
| TL (mm) | 18.69±0.19 | 18.53±0.18 | 18.43±0.2 | 18.58±0.18 |
| HW/TL(mg/mm) | 6.63±0.16 | 6.8±0.08 | 10.35±0.13* | 11.72±0.29*† |
| LVW(mg) | 94.13±2.67 | 94.4±0.75 | 150.1±2.47* | 172.67±4.27*† |
| LVW/TL(mg/mm) | 5.04±0.15 | 5.1±0.05 | 8.15±0.14* | 9.29±0.21*† |
| HR(bmp) | 474±9 | 485±9 | 465±10 | 453±6* |
| IVSd(mm) | 0.64±0.02 | 0.64±0.02 | 0.94±0.02* | 1.05±0.02*† |
| IVSs(mm) | 0.96±0.01 | 0.97±0.02 | 1.24±0.03* | 1.31±0.02* |
| LVIDd(mm) | 3.16±0.06 | 3.19±0.03 | 3.88±0.04* | 4.09±0.05*† |
| LVIDs(mm) | 1.85±0.07 | 1.86±0.03 | 2.87±0.05* | 3.17±0.04*† |
| LVPWd(mm) | 0.66±0.02 | 0.65±0.02 | 0.94±0.02* | 1.03±0.03*† |
| LVPWs(mm) | 1.01±0.02 | 1.02±0.03 | 1.22±0.02* | 1.32±0.02*† |
| FS(%) | 41.44±1.13 | 40.84±0.8 | 26.05±0.77* | 22.45±0.55*† |
| EF(%) | 73.62±1.3 | 73.00±0.98 | 53.27±1.17* | 47.78±0.84*† |
| E/e’ | 20.77±0.23 | 21.5±0.74 | 35.05±0.81* | 38.64±0.72*† |

Body weight(BW), Heart weight(HW), Left ventricular weight(LVW), Lung weight(LW), Tibial length(TL), Heart rates(HR), Interventricular end-diastolic septum thickness(IVSd), Interventricular end-systolic septum thickness (IVSs), Left ventricular end-diastolic internal diameter (LVIDd), LV end-systolic internal diameter (LVIDs), LV end-diastolic posterior wall thickness(LVPWd), LV end-systolic posterior wall thickness (LVPWs), Fractional shortening (FS), Ejection fraction(EF), The ratio of peak early transmitral flow velocity to the peak early diastolic mitral annular velocity(E/e’), transverse aortic constriction (TAC). Data are presented as mean ± SEM. Statistical significance was determined by Two-way ANOVA with interaction analysis and Tukey or Bonferroni post-test. **P*<0.05 compared to SHAM/Ctrl group; ^†^*P* <0.05 compared to TAC/Ctrl group.

**Supplementary Table 5.** Echocardiographic and anatomical assessments of mice with QRICH1 knockdown and ectopic ATF6 expression compared to non-transgenic controls (Ctrl) following 4 weeks of ISO administration

| Groups（ISO） | Ctrl | Ctrl  +ATF6 | QRICH1 KD | QRICH1 KD +ATF6 |
| --- | --- | --- | --- | --- |
| Number | n=10 | n=11 | n=9 | n=10 |
| BW (g) | 27.15±0.29 | 27.18±0.22 | 27.03±0.28 | 27.18±0.28 |
| HW (mg) | 130.8±1.1 | 136.64±1.2 | 118.89±1.09* | 133.3±1.16† |
| HW/BW(mg/g) | 4.82±0.04 | 5.03±0.02 | 4.4±0.04* | 4.9±0.04† |
| LW (mg) | 129.2±1.79 | 138.82±1.17 | 126.56±2.3 | 130.9±1.72† |
| LW/BW(mg/g) | 4.76±0.08 | 5.11±0.05 | 4.69±0.09 | 4.8±0.08† |
| TL (mm) | 19.2±0.2 | 18.68±0.14 | 18.72±0.17 | 19.4±0.2 |
| HW/TL(mg/mm) | 6.8±0.09 | 7.32±0.09 | 6.36±0.09* | 6.9±0.08† |
| LVW(mg) | 105.5±1.28 | 110.36±0.85 | 93.11±1.5* | 108.2±1.1† |
| LVW/TL(mg/mm) | 5.5±0.11 | 5.91±0.08 | 4.97±0.07* | 5.58±0.09† |
| HR(bmp) | 485±3 | 477±5 | 479±7 | 485±3 |
| IVSd(mm) | 0.77±0.01 | 0.82±0.01 | 0.64±0.02* | 0.77±0.01† |
| IVSs(mm) | 1.1±0.03 | 1.16±0.01 | 0.97±0.01* | 1.1±0.03† |
| LVIDd(mm) | 3.5±0.03 | 3.81±0.03 | 3.18±0.04* | 3.5±0.03† |
| LVIDs(mm) | 2.11±0.02 | 2.43±0.03 | 1.86±0.02* | 2.11±0.02† |
| LVPWd(mm) | 0.78±0.01 | 0.84±0.01 | 0.67±0.02* | 0.78±0.01† |
| LVPWs(mm) | 1.11±0.03 | 1.17±0.01 | 0.98±0.01* | 1.11±0.03† |
| FS(%) | 39.71±0.3 | 35.03±1.25 | 41.43±0.34* | 39.71±0.3† |
| EF(%) | 71.64±0.36 | 67.27±0.65 | 73.68±0.45* | 71.64±0.36† |
| E/e’ | 21.08±0.53 | 25.82±0.56 | 20.06±0.45 | 21.08±0.53† |

Body weight(BW), Heart weight(HW), Left ventricular weight(LVW), Lung weight(LW), Tibial length(TL), Heart rates(HR), Interventricular end-diastolic septum thickness(IVSd), Interventricular end-systolic septum thickness (IVSs), Left ventricular end-diastolic internal diameter (LVIDd), LV end-systolic internal diameter (LVIDs), LV end-diastolic posterior wall thickness(LVPWd), LV end-systolic posterior wall thickness (LVPWs), Fractional shortening (FS), Ejection fraction(EF), The ratio of peak early transmitral flow velocity to the peak early diastolic mitral annular velocity(E/e’), isoproterenol (ISO). Data are presented as mean ± SEM. Statistical significance was determined by Two-way ANOVA with interaction analysis and Tukey or Bonferroni post-test. **P*<0.05 compared to Ctrl group; ^†^*P* <0.05 compared to QRICH1 KD group.

**Supplementary Table 6.** List of prime sequences used in this study.

| Species | Gene | sense | antisense |
| --- | --- | --- | --- |
| Human | *Qrich1* | GGTCACTCCACTTGGCTATGTCT | GCTGGTCCACTGTCTTCAATAGG |
| Human | *Gapdh* | CATGTTCGTCATGGGTGTGAA | GGCATGGACTGTGGTCATGAG |
| Rat | *Qrich1* | GCCAGCAGATTCAAATTCAGAC | GTCCACTTTGCGTTTCTTCAC |
| Rat | *Gapdh* | ATGCGGTTTCTAGGTTCACG | ATGTTTTCTGGGGTGCAAAG |
| Rat | *Anp* | CTTCTCCATCACCAAGGGCTTCTTC | TCCAGGTGGTCTAGCAGGTTCTTG |
| Rat | *Bnp* | CCAGTCTCCAGAACAATCCACGATG | GCCTTGGTCCTTTGAGAGCTGTC |
| Rat | *β-mhc* | CACCAGCCTCATCAACCAGAAGAAG | TCCTCTGCGTTCCTACACTCCTG |
| Rat | *Atf6-1* | TGAATTCTGACGCGGCTTTCC | AAATGGTGAGTGAACGCGGATC |
| Rat | *Atf6-2* | TTGCACGTGTATATGCGGATGC | TCCTCGTGGCTGATGTTACTACG |
| mouse | *Qrich1* | CAGTGTTCAACCACAAACTCAG | CAGAGATGGAGTCTGAATGGAG |
| mouse | *Gapdh* | CACGGCAAATTCAACGGCACAG | AGACACCAGTAGACTCCACGACATAC |
| mouse | *Anp* | AGGCCATATTGGAGCAAATCC | GCTTCCTCAGTCTGCTCACTCA |
| mouse | *Bnp* | CTGTCCCAGATGATTCTGTTTCTG | GGCCATTTCCTCCGACTTTT |
| mouse | *β-mhc* | GGAGGCTCTGATCTCTCAGCTAA | GTTCCCTCAGCAGGTCACAATC |

**Supplementary Table 7.**

STROBE Statement—Checklist of items that should be included in reports of ***case-control studies***

|  | Item No | Recommendation | Page No |
| --- | --- | --- | --- |
| **Title and abstract** | 1 | (*a*) Indicate the study’s design with a commonly used term in the title or the abstract | 1 |
|  |  | (*b*) Provide in the abstract an informative and balanced summary of what was done and what was found | 2 |
| Introduction | | | |
| Background/rationale | 2 | Explain the scientific background and rationale for the investigation being reported | 3 |
| Objectives | 3 | State specific objectives, including any prespecified hypotheses | 3 |
| Methods | | | |
| Study design | 4 | Present key elements of study design early in the paper | 4,  Supplementary  Methods |
| Setting | 5 | Describe the setting, locations, and relevant dates, including periods of recruitment, exposure, follow-up, and data collection | Supplementary  Methods, Supplemental Table S1 |
| Participants | 6 | (*a*) Give the eligibility criteria, and the sources and methods of case ascertainment and control selection. Give the rationale for the choice of cases and controls | Supplementary  Methods |
|  |  | (*b*) For matched studies, give matching criteria and the number of controls per case | N/A |
| Variables | 7 | Clearly define all outcomes, exposures, predictors, potential confounders, and effect modifiers. Give diagnostic criteria, if applicable | Supplementary  Methods, Supplemental Table S1 |
| Data sources/ measurement | 8* | For each variable of interest, give sources of data and details of methods of assessment (measurement). Describe comparability of assessment methods if there is more than one group | Supplementary  Methods |
| Bias | 9 | Describe any efforts to address potential sources of bias | Supplementary  Methods |
| Study size | 10 | Explain how the study size was arrived at | 4,  Supplementary  Methods |
| Quantitative variables | 11 | Explain how quantitative variables were handled in the analyses. If applicable, describe which groupings were chosen and why | 4,  Supplementary  Methods |
| Statistical methods | 12 | (*a*) Describe all statistical methods, including those used to control for confounding | 4,  Supplementary  Methods |
|  |  | (*b*) Describe any methods used to examine subgroups and interactions | N/A |
|  |  | (*c*) Explain how missing data were addressed | 4,  Supplementary  Methods |
|  |  | (*d*) If applicable, explain how matching of cases and controls was addressed | 4,  Supplementary  Methods |
|  |  | (*e*) Describe any sensitivity analyses | N/A |
| Results | | | |
| Participants | 13* | (a) Report numbers of individuals at each stage of study—eg numbers potentially eligible, examined for eligibility, confirmed eligible, included in the study, completing follow-up, and analysed | Supplementary  Methods |
|  |  | (b) Give reasons for non-participation at each stage | Supplementary  Methods |
|  |  | (c) Consider use of a flow diagram | N/A |
| Descriptive data | 14* | (a) Give characteristics of study participants (eg demographic, clinical, social) and information on exposures and potential confounders | Supplemental Table S1 |
|  |  | (b) Indicate number of participants with missing data for each variable of interest | Supplementary  Methods,  Supplemental Table S1 |
| Outcome data | 15* | Report numbers in each exposure category, or summary measures of exposure | Supplemental Table S1 |

| Main results | | 16 | (*a*) Give unadjusted estimates and, if applicable, confounder-adjusted estimates and their precision (eg, 95% confidence interval). Make clear which confounders were adjusted for and why they were included | 4,  Supplemental Table S1,  Figure 1A, 1C |
| --- | --- | --- | --- | --- |
|  |  |  | (*b*) Report category boundaries when continuous variables were categorized | 4,  Supplemental Table S1,  Figure 1A, 1C |
|  |  |  | (*c*) If relevant, consider translating estimates of relative risk into absolute risk for a meaningful time period | N/A |
| Other analyses | 17 | Report other analyses done—eg analyses of subgroups and interactions, and sensitivity analyses | | N/A |
| Discussion | | | | |
| Key results | 18 | Summarise key results with reference to study objectives | | 9-11 |
| Limitations | 19 | Discuss limitations of the study, taking into account sources of potential bias or imprecision. Discuss both direction and magnitude of any potential bias | | 12 |
| Interpretation | 20 | Give a cautious overall interpretation of results considering objectives, limitations, multiplicity of analyses, results from similar studies, and other relevant evidence | | 9-12 |
| Generalisability | 21 | Discuss the generalisability (external validity) of the study results | | 11-12 |
| Other information | | | | |
| Funding | 22 | Give the source of funding and the role of the funders for the present study and, if applicable, for the original study on which the present article is based | | 13 |

*Give information separately for cases and controls.

**Note:** An Explanation and Elaboration article discusses each checklist item and gives methodological background and published examples of transparent reporting. The STROBE checklist is best used in conjunction with this article (freely available on the Web sites of PLoS Medicine at http://www.plosmedicine.org/, Annals of Internal Medicine at http://www.annals.org/, and Epidemiology at http://www.epidem.com/). Information on the STROBE Initiative is available at http://www.strobe-statement.org.
